# Stability and interaction defects in the Sin3A-PAH1 αα-hub domain associated with loss-of-function variants

**DOI:** 10.1016/j.jbc.2026.111312

**Published:** 2026-02-26

**Authors:** Amanda D. Due, Sigrid Jørsboe, Louise T. Jensen, Charlotte O’Shea, Majken Staulund, Martin Willemoës, Birthe B. Kragelund, Ida M.Z. Sjøgaard, Karen Skriver

**Affiliations:** 1 REPIN, University of Copenhagen, Copenhagen, Denmark; 2 Linderstrøm-Lang Centre for Protein Science, University of Copenhagen, Copenhagen, Denmark; 3 Structural Biology and NMR Laboratory, Department of Biology, University of Copenhagen, Copenhagen, Denmark

**Keywords:** IDP, IDR, protein domain, protein folding, protein–protein interaction, structure–function, transcription coregulator, side-chain variant, disease

## Abstract

Hub proteins organize large interactomes using their hub domains for protein–protein interactions. αα-hub domains are present in transcriptional regulators, such as Switch-insensitive 3A (Sin3A), an integral scaffolding protein of histone deacetylation complexes. Here, we relate αα-hub stability to function by structural and thermodynamic studies of the Sin3A-paired amphipathic helix 1 (PAH1) wt hub domain and two predicted loss-of-function variants (A126V and K155E) found in patients with the neurodevelopmental Witteveen–Kolk syndrome. For an αα-hub domain, the PAH1 domain is relatively stable with a Δ*G*_DN_ of 14.1 ± 0.9 kJ mol^−1^, which likely compromises its adaptability in binding, thereby increasing specificity. For the two known binding partners, ten–eleven translocase 1 (Tet1) and Sin3A-associated protein 25 (SAP25), a sequence motif undergoes coupled folding and binding with Sin3A-PAH1. The SAP25 interaction shows prototypical thermodynamics for a disorder-based interaction with an enthalpy-driven interaction counteracted by an entropic penalty. In contrast, for the Tet1 interaction, there is no net contribution from entropy to binding, even when including a disordered context in the motif-containing Tet1 fragment. The variants of Sin3A-PAH1 exhibit lower affinities for both SAP25 and Tet1, with a >15-fold reduction in the affinity of PAH1-K155E for Tet1. Notably, the A126V side-chain substitution results in an increased global stability (ΔΔ*G*_DN_ = 6.9 kJ mol^−1^), representing a case of increased stability associated with loss of function. Our findings show how disease-associated Sin3A-PAH1 variants can affect global domain stability and flexibility as well as interactions with specific partner proteins, thereby contributing to the deconvolution of signal fidelity governed by folded hubs in interactions with disordered partner proteins.

Hub proteins form highly connected protein networks central to the efficient regulation of cellular signaling and are omnipresent in the organism. In the nucleus, modification of chromatin constitutes an important regulatory step in gene transcription. Hub proteins like Switch-insensitive 3 (Sin3) can assemble complexes with histone deacetylase (HDAC) enzymes and other proteins, including transcription factors (TFs), to modify chromatin (1, 2, 3). Sin3, like other coregulators, interacts with numerous unrelated TFs and *vice versa* (4). For these interactions, the TFs often use their intrinsically disordered regions (IDRs), which are characterized by low-complexity features and a low degree of sequence conservation (5, 6). How hub domains govern specificity and selectivity in these large networks remains mostly elusive.

The αα-hubs are small, folded domains of app. 70 residues defined by a core αα-hairpin supersecondary structure motif connected to additional α-helices, making up a hydrophobic binding pocket. αα-hubs are present in important human and plant transcriptional regulators, such as Sin3, radical-induced cell death 1 (RCD1), TATA-box binding protein–associated factor 4 (TAF4), and CREB-binding protein (7, 8). Low sequence similarities of the αα-hubs allow for differences in malleability and stability, which, together with the number of helices, are likely determinants of partner binding specificity (7, 9). As a scaffold protein in HDAC complexes, Sin3A regulates gene expression through chromatin modulation (1). The scaffolding properties of Sin3A reside within the three paired amphipathic helix 1 to 3 (PAH1–3) domains, which are all αα-hub domains, and a HDAC interaction domain (Fig. 1*A*) (1). Due to its association with HDACs, Sin3A has been linked to negative regulation of gene expression, and, accordingly, deletion of *Sin3A* in yeast results in the upregulation of more than 170 genes (10). However, the same deletion also results in downregulation of more than 200 genes, indicative of both negative and positive regulation (10). Functionally, Sin3A–HDAC complexes are implicated in numerous processes, including in the central nervous system (11), cell differentiation, and apoptosis, and thus, play a role in the development of cancers (12).

**Figure 1:**
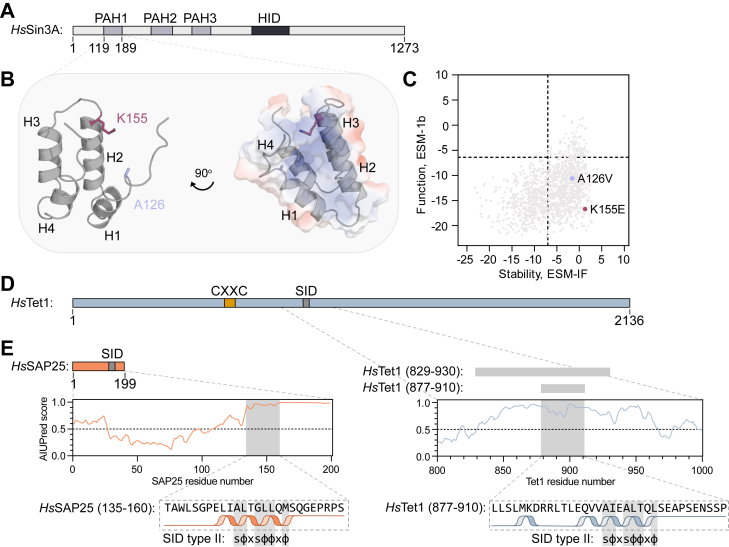
**Domain architecture and structure of Sin3A and its interaction partners Tet1 and SAP25.***A*, *Homo sapiens* (*Hs*) Sin3A contains several folded domains within a long disordered context. *B*, *left*, PDB structure 2RMR (14) of PAH1 with its four helices labeled H1–H4. Positions corresponding to the missense mutations A126V and K155E associated with Witteveen–Kolk syndrome are shown as *sticks* (A126, *lilac*; K155, *red*). *Right*, the Sin3A-PAH1 structure with an electrostatic surface potential representation, with negative and positive charges shown in *red* and *blue*, respectively. *C*, data extracted from the study by Cagiada *et al.* (15), which predicts stability *versus* functionality of all possible mutations in PAH1. Here, we show ESM-1b, which is a protein language model to predict variant effects on protein function, and ESM-IF, which is a combined neural network and language model predicting variant effects on protein stability. Locations of A126V and K155E are highlighted. *Zero* indicates wt-like predictions, and *lines* indicate thresholds determined using Youden’s index (15). Negative values indicate either decreased stability or loss of function compared with wt. *D*, *top*, domain architecture of Tet1. *Bottom*, disorder prediction by AIUPred (37) of the region containing the SID *(gray*) in Tet1, with the Tet1 fragments used in this study shown above and the SID sequence of Tet1 and the SID consensus sequence below the prediction. *E*, *top*, domain architecture of SAP25. *Bottom*, disorder prediction by AIUPred (37) of the region containing the SID (*gray*) in SAP25 with the SID sequence of the SAP25 fragment used in this study and the SID consensus sequence shown below the prediction. Helix representation according to the AlphaFold3 (36) PAH1-wt–SAP25 and PAH1-wt–Tet1 models (14, 17). The domain architecture diagrams are drawn to scale. H1–H4, helix 1–4; PAH1, paired amphipathic helix 1; PDB, Protein Data Bank; SAP25, Sin3A-associated protein 25; Sin3A, Switch-insensitive 3A; Tet1, ten–eleven translocase 1; s, aliphatic residue; SID, Sin3-interacting domain; ϕ, hydrophobic residue; x, any residue except proline.

Heterozygous deletions, nonsense, and missense mutations in *Sin3A* were suggested as the causes of the neurodevelopmental Witteveen–Kolk syndrome (13). Patients have delayed cognitive and motoric development as well as distinct facial features and short stature. One of the missense mutations leads to the Ala126Val side-chain variant at a position just before and within helix 1 (H1) of unbound and partner-bound Sin3A-PAH1, respectively (14). For the Lys155Glu variant, the substitution is in H2 of the four α-helix PAH1 domains (Fig. 1*B*) (13, 14), with H2 forming one of the helices of the αα-hairpin. According to a recent machine learning study that predicts functional and stability effects of all missense mutations in human proteins, the two mutations have a negative impact on function but no effect on stability (Fig. 1*C*) (15).

PAH1 of Sin3A has numerous interaction partners (7), including the enzyme ten–eleven translocase 1 (Tet1) involved in DNA demethylation (16, 17), and HDAC complex subunit Sin3A-associated protein 25 (SAP25) (17), which functions as an adaptor, recruiting various enzymatic components to Sin3–HDAC complexes (Fig. 1, *D* and *E*) (18). The regions responsible for the interactions with PAH1 are intrinsically disordered and share a sequence motif known as the Sin3-interacting domain (SID) (Fig. 1, *D* and *E*) (19). The motif consists of hydrophobic residues and is described as type I or II, which are reversed versions of each other (20). NMR studies showed that SID-containing peptides derived from Tet1 and SAP25 form an α-helix upon binding to PAH1 (14, 17), resulting in a bound state that is shared with other PAH binding partners (21, 22, 23). The conserved hydrophobic residues of the SID of Tet1 and SAP25 point toward the open-end hydrophobic binding cleft of PAH1 (14, 17), primarily located between H1 and H2 (Fig. 1*B*).

Here, we address the effects of αα-hub structure and malleability on interactions using Sin3A-PAH1 as a model. We characterize the stability of Sin3A-PAH1 and the thermodynamics of its interactions with Tet1 and SAP25. By comparing the structures, stabilities, and interactions for Sin3A-PAH1 and the A126V and K155E variants, we discuss how the changes may lead to Witteveen–Kolk syndrome (13). Whereas Sin3A-PAH1–A126V exhibits both increased stability and decreased affinities for the interaction partners compared with Sin3A-PAH1–wt, Sin3A-PAH1–K155E is only affected in its interactions. Together, our results suggest that PAH1 is a relatively stable and less malleable αα-hub domain, with stability empowering partner selectivity in interactomes, although rigidity may also abolish interactions. The study thus contributes to the deconvolution of signal fidelity governed by folded hubs in their interactions with intrinsically disordered ligands.

## Results

### Sin3A-PAH1 is relatively stable, and the A126V substitution further increases stability

**Figure 2:**
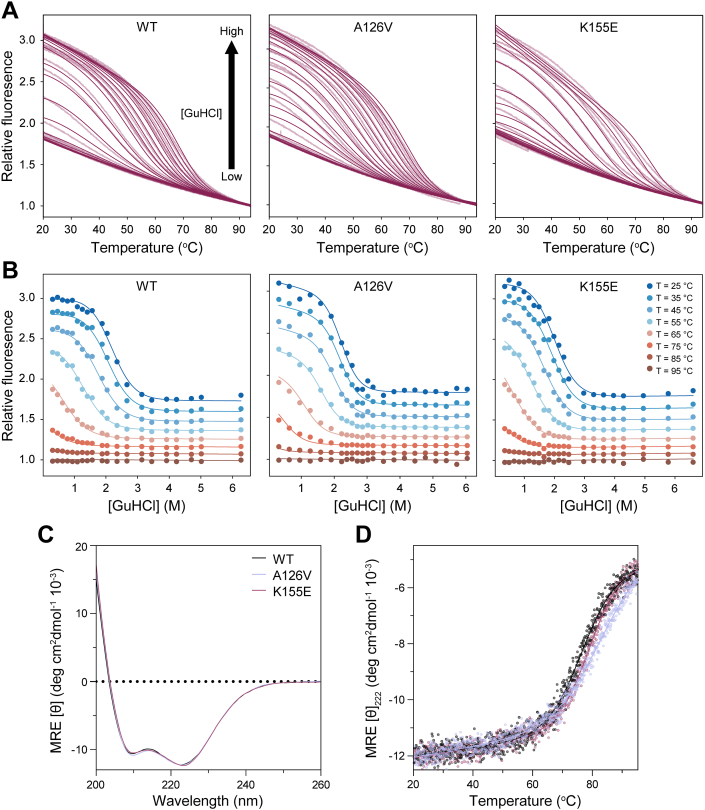
**Unfolding of PAH1_115–212_ variants by denaturant and temperature.***A* and *B*, nanoDSF experiment with two-dimensional unfolding for denaturant (*A*) and temperature (*B*) by measuring internal fluorescence at 330 nm and fitting to Equation 3 (see the Experimental procedures section) for each PAH1_115–212_ variant. *A*, the experimental data are shown in *light purple* for 0.2 to 6.5 M GuHCl concentrations as indicated by the *arrow*, and *fits* are shown as *dark purple lines*. *B*, denaturation curves were extracted at eight different temperatures from *A*. The data are shown as *dots*, whereas the fits are shown as *lines*. Triplicates were made for each variant, and the data and fits of the replicates are shown in Figs. S2 and S3. *C*, CD spectra with mean residue ellipticity (MRE) as a function of wavelength for the PAH1_115–212_ variants. *D*, temperature denaturation of the PAH1_115–212_ variants measured by CD spectroscopy using the MRE at 222 nm. The data *(dots*) were fitted to Equation 2 (*solid line*). GuHCl, guanidine hydrochloride; nanoDSF, nano differential scanning fluorimetry; PAH1, paired amphipathic helix 1.

The stability of folded domains has been linked to the size of their interactome, as also suggested for the αα-hubs (9), making stability studies of the αα-hubs highly relevant. We analyzed the stability of the wt PAH1 domain of Sin3A (*Hs*PAH1_115–212_, referred to as PAH1_115–212_–wt) and the PAH1 domain variants; PAH1_115–212_–A126V and PAH1_115–212_–K155E. In addition to the αα-hub domain, these fragments contain a short C-terminal β-sheet making contacts with the α-helices, possibly affecting the stability of PAH1 (Fig. S1). We performed combined chemical (guanidine hydrochloride [GuHCl]) and temperature unfolding experiments on the PAH1_115–212_ variants using nano differential scanning fluorimetry (nanoDSF) and measured changes in internal fluorescence at 350 and 330 nm. As PAH1_115–212_ is devoid of tryptophan residues, we focused on the fluorescent signal at 330 nm. The data were analyzed using a two-dimensional fitting procedure (24), accounting for both temperature- and denaturant-dependent unfolding. The relative fluorescence is shown as a function of temperature (Fig. 2*A*) and denaturant concentration (Fig. 2*B*), and the resulting parameters are presented in Table 1. The plots suggest two-state unfolding for all PAH1_115–212_ variants for both temperature- and denaturant-dependent unfolding.

**Table 1:** Stability of PAH1 variants determined by nanoDSF and CD spectroscopy

Variant	*T*_m_ (°C)	Δ*H* (kJ mol^−1^)^a^	Δ*C*_p_ (kJ mol^−1^ K^−1^)	*m* (kJ mol^−1^ M^−1^)	Δ*G*_DN_ (kJ mol^−1^)	ΔΔ*G*_DN_ (kJ mol^−1^)
PAH1_115–212_–wt						
Two-dimensional unfolding^b^	71 ± 1	190 ± 10	3.6 ± 0.3	6.96 ± 0.1	14.1 ± 0.9	—
CD thermal denaturation	76.5 ± 0.9	170.2 ± 0.7	—	—	—	—
PAH1_115–212_–A126V						
Two-dimensional unfolding^b^	80.5 ± 0.3	283 ± 7	5.1 ± 0.2	9.0 ± 0.3	21.0 ± 0.3	−6.9 ± 0.4
CD thermal denaturation	83 ± 3	131.2 ± 0.6	—	—	—	—
PAH1_115–212_–K155E						
Two-dimensional unfolding^b^	72 ± 2	195 ± 5	3.8 ± 0.2	7.0 ± 0.6	13.9 ± 0.2	0.2 ± 0.3
CD thermal denaturation	78.9 ± 0.8	188.4 ± 0.7	—	—	—	—

a Δ*H* corresponds to the Δ*H*_vH_ for the CD thermal denaturation and Δ*H*_m_ for two-dimensional global analysis.

b Parameters are given as the mean of triplicate measurements with a standard deviation.

The two-dimensional unfolding analysis revealed a melting temperature (*T*_m_) for PAH1_115–212_–wt of 71 ± 1 °C and a free energy of unfolding (Δ*G*_DN_) of 14.1 ± 0.9 kJ mol^−1^ (Table 1). For PAH1_115–212_–K155E, we obtained a *T*_m_ and a Δ*G*_DN_ of 72 ± 2 °C and 13.9 ± 0.2 kJ mol^−1^, similar to the values for PAH1_115–212_–wt. Hence, with ΔΔ*G*_DN_ = 0.2 ± 0.3 kJ mol^−1^, the mutation at position 155 does not affect the overall structural stability of PAH1, even though the charge is reversed. In contrast, for the A126V substitution (PAH1_115–212_–A126V), *T*_m_ increased significantly to 80.5 ± 0.3 °C, whereas Δ*G*_DN_ increased to 21.0 ± 0.3 kJ mol^-1^, with all parameters (*T*_m_, Δ*H*_m_, Δ*C*_p_, *m*, and Δ*G*_DN_) (Table 1) being higher than for PAH1_115–212_–wt and PAH1_115–212_–K155E. This suggests that the A126V side-chain substitution located four residues before H1 of unbound PAH1 (Fig. 1*B*) stabilizes the structure of the domain, as evident from ΔΔ*G*_DN_ = −6.9 ± 0.4 kJ mol^−1^. Δ*C*_p_ and *m* are related to the change in accessible surface area upon unfolding (25), with the average Δ*C*_p_ reported to be 0.06 kJ mol^−1^ K^−1^ per residue for the transition of buried to exposed surface area (25). Using this value, 61, 64, and 86 residues within PAH1_115–212_–wt, –K155E, and –A126V, respectively, become exposed upon unfolding. The difference observed for PAH1_115–212_–A126V can be explained either by a more folded state or a more unfolded state for the variant.

To investigate if the side-chain substitutions change the amount of secondary structure within PAH1, we recorded a far-UV CD spectrum for each PAH1_115–212_ variant (Fig. 2*C*). All spectra exhibited a profile characteristic of α-helical structure, with minima at 208 and 222 nm, and only minor differences between the spectra, suggestive of a similar amount of α-helical structure in the PAH1_115–212_ variants. Also using CD spectroscopy, we performed thermal denaturation of the PAH1_115–212_ variants and calculated the mean residue ellipticity at 222 nm (Fig. 2*D*). Measuring thermal denaturation between 20 and 95 °C resulted in curves fitting best to a two-state unfolding process in agreement with the above-described two-dimensional unfolding analysis. However, the slope of the transition varied among the PAH1_115–212_ variants, and for PAH1_115–212_–A126V, a plateau was not fully reached for the unfolded state. From the fitted data, the *T*_m_s were determined to be 76.5 ± 0.9, 83 ± 3, and 78.9 ± 0.8 °C for PAH1_115–212_–wt, –A126V, and –K155E, respectively (Table 1). Thus, the *T*_m_ values determined follow the same trend as for the two-dimensional unfolding analysis. The Δ*H*_m_s determined from CD spectroscopy and two-dimensional analysis revealed different trends, with Δ*H*_m_ for PAH1_115–212_–A126V being low (131.2 ± 0.6 kJ mol^−1^). The thermal denaturation followed by CD spectroscopy monitors the change in α-helical secondary structure, whereas the two-dimensional unfolding analysis measures the change in tertiary structure. This may explain the differences in the values for the parameters (*T*_m_ and Δ*H*_m_) obtained by the two methods. Overall, the CD experiment suggests that the PAH1_115–212_ variants differ with respect to stability, with PAH1_115–212_–A126V being more stable based on the higher *T*_m_ obtained in both experiments (Fig. 2*D*).

### Changed dynamics of PAH1–A126V

**Figure 3:**
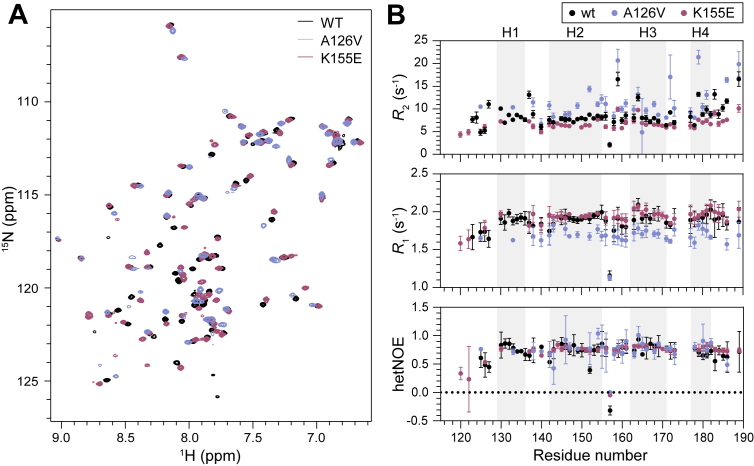
**Fast time scale backbone dynamics of the PAH1_119–189_ variants.***A*, ^1^H–^15^N-HSQCs of 100 μM of PAH1_119–189–_wt, –A126, or –K155E at 30 °C and pH 7.4. *B*, transverse relaxation rates (*R*_2_) (*top*), longitudinal relaxation rates (*R*_1_) (*middle*), and ^1^H–^15^N-hetNOEs (*bottom*) for the PAH1_119–189_ variants. Standard error of the fit is included for *R*_2_ and *R*_1_ at each residue position. Mean and standard deviation of triplicates are shown for hetNOEs. Color codes for *A* and *B* are shown in the *upper right corner* of each subfigure. hetNOE, heteronuclear NOE; HSQC, heteronuclear single quantum coherence; PAH1, paired amphipathic helix 1.

To further address the stabilities of the PAH1 variants, we investigated their fast timescale backbone dynamics using NMR spectroscopy. PAH1_115–212_ used in stability studies had a ^1^H,^15^N-heteronuclear single quantum coherence (HSQC) spectrum that was different from that of a shorter previously assigned PAH1_119–189_ fragment from mouse (14). Human PAH1_119–189_ is identical to mouse PAH1_119–189_ at the amino acid sequence level (14). Therefore, we recorded ^1^H,^15^N-HSQCs of each PAH1_119–189_ variant (Fig. 3*A*), and the reported assignments of mouse PAH1_119–189_ (14) were transferred to the recorded spectra (Fig. S4). However, we chose buffer conditions (pH 7.4) and a temperature (30 °C) that are closer to physiological conditions. Hence, the assignment could not be transferred directly, and pH and temperature titrations were conducted on PAH1_119–189_–wt to assist the assignment transfer (Figs. S5 and S6). Increasing pH did lead to the disappearance of some peaks, and 59 of the 65 residues originally assigned for PAH1_119–189_–wt were assigned here. The pH change resulted in the disappearance of peaks corresponding to four residues in the N-terminal region, and the last two disappearing peaks belong to residues in the linkers between H1 and H2 and H3 and H4 (Fig. 1*B*). For the mutant PAH1 domains, peak positions changed, suggestive of altered chemical environments, making assignment transfer more difficult. For the PAH1_119–189_–K155E, 45 of 65 residues could be assigned. For PAH1_119–189_–A126V, we experienced an additional loss of peaks resulting from line broadening compared with PAH1_119–189_–wt, leading to the assignment of 38 of 65 residues.

We recorded transverse (*R*_2_) and longitudinal (*R*_1_) relaxation rates as well as steady-state ^1^H–^15^N-heteronuclear NOEs (hetNOEs) to compare the dynamics of the three PAH1 variants (Fig. 3*B*). For PAH1_119–189_–wt, the relaxation rates and ^1^H–^15^N-hetNOEs have similar values across the four helices (14). Sahu *et al.* (14) reported that upon partner binding, PAH1 undergoes local folding involving E124–Y129 before H1 and N183–T184 after H4. This is in accordance with the variation in the *R*_2_ rates for the E124–Y129 region just before H1 in the unbound form of PAH1–wt (Fig. 3*B*). The *R*_2_ rates increase for the C-terminal residues after H4, suggesting less flexibility than expected for an unstructured region. The same pattern appears from the hetNOE values, which remain high for the C-terminal residues, similar to the structured parts of PAH1 (Fig. 3*B*). PAH1_119–189_–K155E showed lower *R*_2_ rates across the domain compared with PAH1_119–189_–wt but similar *R*_1_ rates. Larger changes were observed for PAH1_119–189_–A126V, where the *R*_2_ rates increased compared with the other PAH1_119–189_ variants, in accordance with the line broadening resulting in peak loss in the ^1^H–^15^N-HSQC spectrum (Fig. 3*A*). In contrast, the *R*_1_ rates were lower throughout PAH1_119–189_–A126V. As both *R*_2_ and *R*_1_ rates are sensitive to different relaxation processes, we plotted *R*_1_*R*_2_ to estimate the effect of chemical exchange only (26) (Fig. S7). *R*_1_*R*_2_ for residues along the sequence of PAH1_119–189_–wt and –K155E were similar, although with slightly lower *R*_1_*R*_2_ for K155E (Fig. S7). However, *R*_1_*R*_2_ was elevated for PAH1_119–189_–A126V, suggestive of an increased chemical exchange contribution to the relaxation. Analytical size-exclusion chromatography (SEC) was in accordance with the three PAH1_119–189_ variants being monomers and revealed similar elution volumes for all three variants, suggesting they have a similar hydrodynamic radius (Fig. S8). Even though the *R*_2_ and *R*_1_ rates suggest altered chemical exchange of PAH1_119–189_–A126V, the similar ^1^H–^15^N-hetNOEs across the variants indicate that the main-chain rigidity is maintained in the variants.

### Different thermodynamics of the PAH1 interactions with Tet1 and SAP25

**Figure 4:**
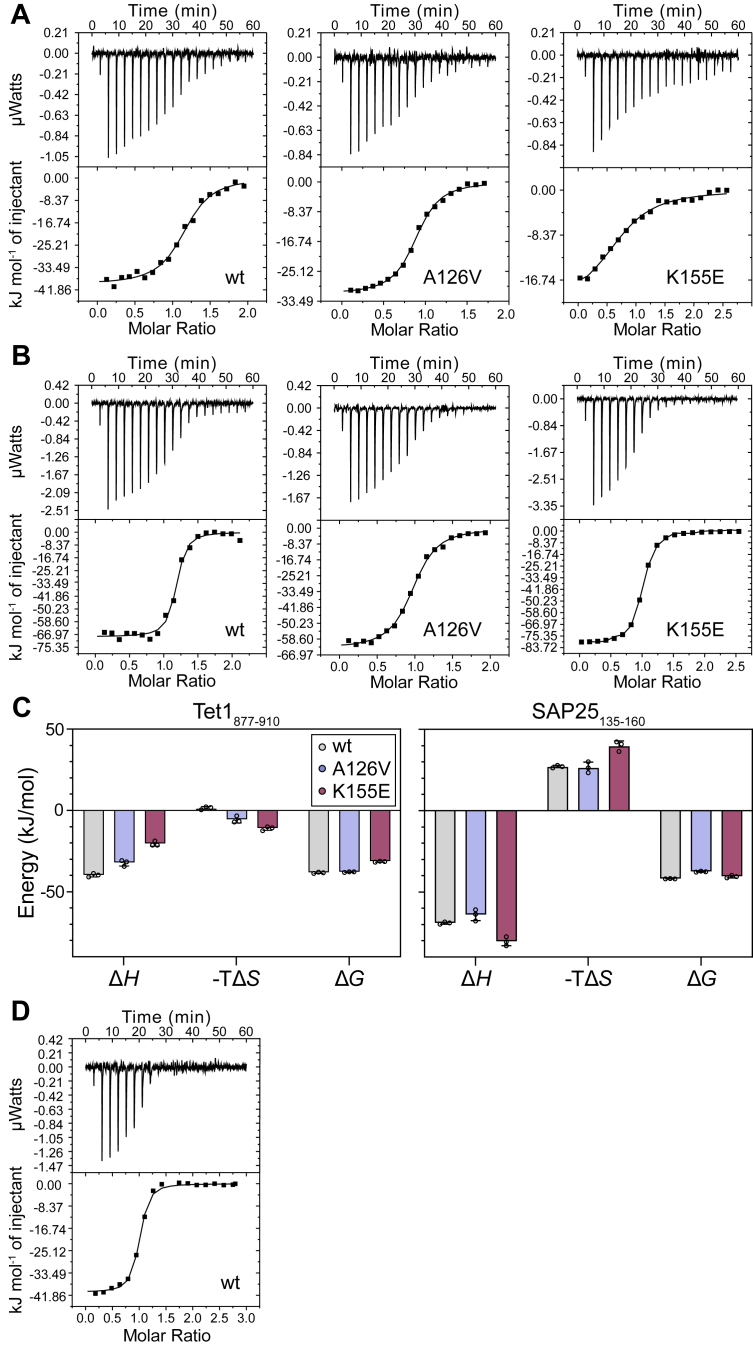
**Thermodynamics of PAH1 variant interactions.***A* and *B*, ITC experiments of PAH1_115–212_–wt (*left*), PAH1_115–212_–A126V (*middle*), and PAH1_115–212_–K155E (*right*) interactions with Tet1_877–910_ (*A*) and SAP25_135–160_ (*B*). *C*, thermodynamic parameters for the interactions of the PAH1 variants with Tet1_877–910_ (*left*) and SAP25_135–160_ (*right*). *Bars* represent the mean with the standard deviation of triplicates. Underlying raw data are shown in Figs. S9 and S10. *D*, ITC experiment of PAH1_115–212_–wt and Tet1_829–930_. In (*A*, *B*, and *D*), each experiment is representative of one of the triplicate experiments (Figs. S9 and S10) used for calculating mean parameters in Table 2, recorded with the thermogram shown on *top* and the fit shown *below*. ITC, isothermal titration calorimetry; PAH1, paired amphipathic helix 1; SAP25, Sin3A-associated protein 25; Tet1, ten–eleven translocase 1.

To characterize the thermodynamics of the PAH1 interactions, we used isothermal titration calorimetry (ITC). We focused on the interactions with the SIDs of Tet1 and SAP25 (Fig. 1*E*), as these are structurally characterized (14, 17). To represent human SAP25, we designed a peptide, SAP25_135–160_, covering the sequence that forms an α-helix upon binding (14). For the interaction with Tet1, we used Tet1_877–910_, previously used for structure characterization (17). ITC directly determines the dissociation constant, *K*_*d*_, the stoichiometry N, and the change in binding enthalpy (Δ*H*) (Figs. 4, *A* and *B*, S9 and S10) and therefore enables the calculation of the change in both Gibbs free energy (Δ*G*) and entropy (Δ*S*) upon binding (Table 2 and Fig. 4*C*).

**Table 2:** Thermodynamic parameters of PAH1 variant interactions with Tet1 and SAP25 peptides

Peptide	PAH1_115–212_ variant	N	*K*_d_ (nM)	Δ*H* (kJ mol^−1^)	−TΔ*S* (kJ mol^−1^)	Δ*G* (kJ mol^−1^)
Tet1_877–910_	wt	1.1 ± 0.1	250 ± 30	−39.8 ± 0.9	1.5 ± 0.8	−38.3 ± 0.3
	A126V	0.88 ± 0.02	310 ± 20	−32 ± 2	−6 ± 2	−37.8 ± 0.2
	K155E	0.9 ± 0.1	3900 ± 500	−20 ± 1	−11 ± 1	−31.4 ± 0.3
SAP25_135–160_	wt	1.0 ± 0.1	63 ± 3	−69.1 ± 0.5	27.2 ± 0.6	−41.8 ± 0.1
	A126V	0.92 ± 0.07	360 ± 40	−64 ± 3	27 ± 3	−37.4 ± 0.2
	K155E	0.97 ± 0.02	120 ± 10	−81 ± 2	41 ± 1	−40.2 ± 0.4
Tet1_829–930_	wt	0.92 ± 0.1	90 ± 10	−40 ± 1	−1 ± 1	−40.9 ± 0.2

Parameters are given as the mean of three measurements, with the error being the SD of the mean.

We obtained a *K*_*d*_ of 250 ± 30 nM for the PAH1_115–212_–wt–Tet1_877–910_ interaction and a *K*_d_ of 63 ± 3 nM for the PAH1_115–212_–wt–SAP25_135–160_ interaction. Both interactions are driven by enthalpy. While the interaction with SAP25_135–160_ has a large entropic penalty (-TΔS = 27.2 ± 0.6 kJ mol^−1^), the interaction with Tet1_877–910_ has almost no net contribution from entropy (-TΔS = 1.5 ± 0.8 kJ mol^−1^) (Fig. 4*C* and Table 2). The change in entropy upon binding is the result of opposite contributions from conformational restrictions and release of water and counterions, respectively (27). However, these different entropic effects cannot be distinguished from the ITC recordings conducted here (27). Since the sequence motif context may dramatically affect binding for ID-based interactions (28, 29), we designed and tested a longer Tet1 fragment, Tet1_829–930_, covering the entire disordered region surrounding the SID (Fig. 1*D*). The longer context impacted the affinity positively with a *K*_d_ of 90 ± 10 nM (Table 2, Figs. 4*D* and S9*D*) but only to a minor effect corresponding to a threefold increase in affinity. Interestingly, even when including the disordered context, there was no net entropic contribution to binding.

### Mutant PAH1 variants have changed Tet1 and SAP25 binding profiles

For the PAH1_115–212_ domains harboring the A126V and K155E side-chain substitutions, the affinities decreased to varying degrees (Table 2). The interaction of Tet1_877–910_ with PAH1_115–212_–A126V and PAH1_115–212_–K155E resulted in a *K*_*d*_ of 310 ± 20 nM and 3900 ± 500 nM, corresponding to a decrease in affinity of less than 2-fold and more than 15-fold, respectively. Although the *K*_*d*_ of the interaction of Tet1_877–910_ with PAH1_115–212_–A126V is similar to that of PAH1_115–212_–wt, a less favorable enthalpic and a more favorable entropic contribution were determined for the interaction with PAH1_115–212_–A126V, suggestive of changes in the binding modes with less enthalpy-dependent structure formation. Compared with its binding to PAH1_115–212_–wt, the association of Tet1_877–910_ with PAH1_115–212_–K155E has a smaller enthalpic contribution to binding, which is only partly compensated by a more favorable change in entropy (Table 2 and Fig. 4*C*). The interactions with SAP25_135–160_ were less affected, with a sixfold increase in *K*_*d*_ for PAH1_115–212_–A126V explained by a small change in binding enthalpy and a twofold increase in *K*_*d*_ for PAH1_115–212_–K155E as a result of an increase in binding entropy, which is only partly compensated by an increased binding enthalpy (Table 2 and Fig. 4*C*, *left*).

In conclusion, the thermodynamics of the PAH1_115–212_–wt interactions with Tet1_877–910_ and SAP25_135–160_ are different, with SAP25_135–160_ having a large entropic penalty (Fig. 4*C*). This suggests different bound states of the peptides, even though both SAP25_135–160_ and Tet1_877–910_ undergo coupled folding and binding upon complex formation with PAH1_115–212_–wt. The naturally occurring PAH1 variants have profiles of binding to both SAP25 and Tet1, which are different from those of wt PAH1 and with compromised affinities.

### NMR analysis supports changed interactions of variant PAH1 domains

**Figure 5:**
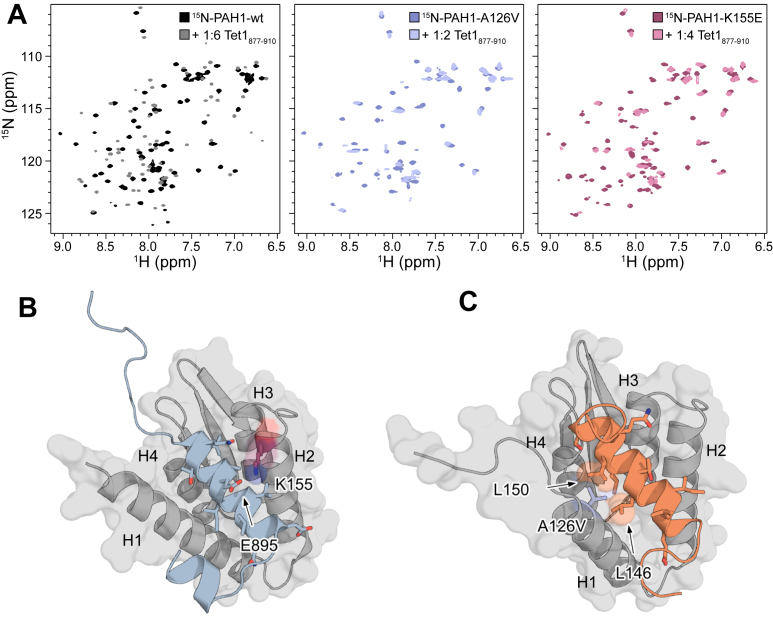
**Changed interactions and structural effects of PAH1 mutant side-chain variants.***A*, superimposed ^1^H–^15^N-HSQC endpoints for 100 μM PAH1_119–189_–wt (*left*), PAH1_119–189_–A126V (*middle*), and PAH1_119–189_–K155E (*right*) with and without the molar ratio of Tet1_877–910_ indicated in each plot (>95% saturation). *B*, AlphaFold3 model of the complex between Tet1_877–910_ (*blue*) and PAH1_119–189_–wt (*gray*), with side chains of α-helix forming Tet1_877–910_ residues shown as *sticks* (36). *C*, AlphaFold3 model of the complex between SAP25_135–160_ (*orange*) and PAH1_119–189_–wt (*gray*) with side chains of α-helix forming SAP25_135–160_ residues shown as *sticks* (36). *Sphere* representation was used for L146 and L150. *B* and *C*, K155 and A126V are highlighted as *sticks*. The substitution A126V was introduced in the AlphaFold3 model using mutagenesis in PyMOL. The five generated AlphaFold3 models of the complexes are shown in Fig. S14. HSQC, heteronuclear single quantum coherence; PAH1, paired amphipathic helix 1; Tet1, ten–eleven translocase 1.

We further investigated the interaction between Tet1_877–910_ and the PAH1 variants using NMR spectroscopy. Tet1_877–910_ was titrated into ^15^N-labeled PAH1_119–189_–wt, –A126V, or –K155E, and ^1^H–^15^N-HSQCs were obtained (Fig. 5*A*). Endpoint ^1^H–^15^N-HSQCs are superimposed in Figure 5*A* for each of the PAH1_119–189_ variants, and the full titration series for all variants is shown in Fig. S11. The resonances of PAH1_119–189_–wt overall experience a slow exchange regime for the interaction with Tet1_877–910_, as previously shown for the PAH1–Tet1 interaction (17). This gives rise to an unbound and bound peak for most residues assigned (Figs. 5*A*, *left*, and S11*A*), which is often suggestive of a high-affinity interaction. To determine which bound peak corresponds to the assigned unbound peak, we performed ZZ-exchange experiments with delays of 100 to 500 ms to obtain crosspeaks between the two states (Fig. S12). From the ZZ-exchange, we were able to assign the bound state for 50 residues in PAH1_119–189_–wt and calculate the chemical shift perturbations (CSPs) of these residues (Fig. S13). The largest CSPs were obtained for H1 and H2, as well as the C-terminal tail of PAH1–wt, in agreement with the predicted AlphaFold3 complex structure (Figs. 5*B* and S14*A*).

For the interactions of the PAH1_119–189_–A126V and –K155E variants with Tet1_877–910,_ the resonances were also in the slow exchange regime, with peaks originating from free and bound states. However, many signals also disappeared (Figs. 5*A* and S11, *B* and *C*), suggesting exchange dynamics originating from intermediate exchange. Thus, the dynamics in the three systems differ.

## Discussion

The αα-hub domains have diverse biological functions but bind ligands with similar ID-based chemical properties (7), making the αα-hubs suitable models for dissecting selectivity and specificity of interactions between folded hub domains and IDRs. We addressed global stability of PAH1_115–212_–wt and found similar values for Δ*G*_DN_ and *T*_m_ as determined for the αα-hub domains *Hs*TAF4-TAFH (9) and the *Hs*Sin3B-PAH1 and -PAH2 (30) (Table 3), whereas the RST αα-hub domains from *At*TAF4 and *At*RCD1 (9), and especially the molten-globule–like nuclear coactivator binding domain (NCBD) αα-hub domain from CREB-binding protein from mucin (31), have lower values for these parameters (Table 3). Thus, the questions remain if structural differences between the αα-hubs can explain these different global stabilities, and if stability and interactome size correlate inversely, as hypothesized (32, 33). The αα-hairpin supersecondary structure motif forms a structurally stable core, onto which various dynamic α-helices are anchored. For RCD1–RST, H4 is stabilized upon ligand binding, but large structural rearrangements do not occur. The TAFH, PAH1, and PAH2 are structurally similar in the free and bound forms, although with a small N-terminal helix extension of PAH1 induced by partner binding (8, 14). Thus, coupled folding and binding may occur to varying degrees in the outer helices of the αα-hubs, but with major rearrangements occurring only in NCBD (31). The low stability of the RST and NCBD domains allows adaptability to numerous partners. For the PAH domains, which, uniquely to the αα-hubs, exist in tandem (Fig. 1*A*), the requirement for structural flexibility may be less, enabling enhanced specificity of each PAH domain (7).

**Table 3:** Global stabilities and melting temperature of αα-hub domains

Domain	Δ*G*_DN_ (kcal mol^−1^)	*T*_m_ (°C)	Reference
*Hs*Sin3A_115–212_–PAH1	14.1 ± 0.9 kJ	71 ± 1	This study
PAH1_115–212_–A126V	21.0 ± 0.3	80.5 ± 0.3	This study
PAH1_115–212_–K155E	13.9 ± 0.2	72 ± 2	This study
*Hs*TAF4-TAFH	12.9 ± 1.4	71 ± 3	(9)
*Hs*Sin3B-PAH1	12.9 ± 0.5	65.7 ± 2	(30)
*Hs*Sin3B-PAH2	10.2 ± 0.5	64.4 ± 0.4	(30)
*At*TAF4-RST	5.9 ± 1.3	66 ± 2	(9)
*At*RCD1-RST	8.8 ± 1.4	59 ± 5	(9)
*Mu*CBP-NCBD	1.5 ± 0.1		(31)

PAH1_115–212_–K155E is about equally stable as PAH1_115–212_–wt, whereas the side-chain substitution in PAH1_115–212_–A126V had a large effect on stability (Table 1). The *T*_m_ for PAH1_115–212_–A126V increased 9.5 °C compared with that of PAH1_115–212_–wt. In addition, the CD-recorded unfolding process still progressed at 95 °C (Fig. 2). The Δ*C*_p_ value determined for the PAH1_115–212_–A126V also stands out, as does the NMR relaxation data compared with those for PAH1_119–189_–wt and PAH1_119–189_–K155E. That most residues in H1 were unassignable because of their absence in the ^1^H–^15^N-HSQC indicates that the dynamics are altered for H1 of PAH1_119–189_–A126V (Fig. 3). Based on the above, we suggest that the increased stability of PAH1_115–212_–A126V may reduce the affinity for some of its interaction partners (Table 2).

The affinities of αα-hubs for different interaction partners have been determined using different biophysical methods (7). For *Mu*Sin3A, the affinity for *Mu*SAP25_126–186_ is 134 nM ± 13 nM (14), corresponding well with our determination of *K*_*d*_ = 63 ± 3 nM for the analogous interactions between human proteins (Table 2). The thermodynamic profile for the Sin3A-PAH1–SAP25_135–160_ interaction reveals prototypical coupled folding and binding being driven by binding enthalpy and counteracted by the change in entropy, likely because of conformational restrictions upon binding (27). For the PAH1–Tet1_878–911_ interaction, the *K*_*d*_ was estimated by NMR spectroscopy to be <100 nM (17). Here, we determined the *K*_*d*_ to be 250 ± 30 nM by ITC. This interaction has a marginal, favorable net contribution from entropy. Inclusion of the disordered SID context (Tet1_829–930_) resulted in only a threefold increase in affinity for PAH1, and entropy had only a minor effect on binding. Thus, the release of water or counterions upon binding likely counteracts the loss in entropy for the Tet1 interaction with PAH1, which mostly depends on the SID region.

The alleles encoding the A126V and K155E side-chain substitutions identified in PAH1 have been found in patients diagnosed with Witteveen–Kolk syndrome (13). Here, we show that both the *K*_*d*_s and the other thermodynamic binding parameters are affected in these PAH1 variants with two well-described interaction partners. A loss of electrostatic interaction between K155 in PAH1 and E895 in Tet1, substituted with a direct repulsion, likely explains the dramatic effects for the PAH1–Tet1 interaction (Figs. 5*B* and S14*A*). For the interaction between PAH1_115–212_–A126V and SAP25_135–160_, a more restricted flexibility of the PAH1 domain, as indicated by the thermal stability studies (Fig. 2 and Table 1), together with a possible steric hindrance introduced by a bulky, hydrophobic valine side chain close to L146 and L150 at the SAP25 binding surface, are reasonable explanations for the decreased affinity of PAH1_115–212_–126V for SAP25_135–160_ (Figs. 5*C* and S14*B*). The increased stability of PAH1_115–212_–A126V likely affects SAP25 binding more than Tet1 binding, because the association with SAP25 is driven by a larger binding enthalpy, suggestive of more bond formation and structuring. Thus, the association with SAP25 may be more dependent on PAH1 flexibility than the association with Tet1. The side-chain substitution in PAH1–A126V does not lead to a changed hydrodynamic volume (Fig. S8), and based on the CD results, it is unlikely to affect the overall α-helix amount; thus, a structural effect is less likely. These results are, however, obtained in the unbound state, and a valine at the N-terminal end of H1 (14) might impede its extension in the bound state. Thus, effects on stability for PAH1–A126V and the interaction of both PAH1 variants with Tet1 and SAP25 can explain, on a molecular level, how side-chain substitutions at positions 126 and 155 affect function (Fig. 1*C*). As we determined weakened affinities for all the PAH1–A126V and –K155E interactions tested, the side-chain substitutions are likely to negatively affect the interactions of Sin3A-PAH1 with several of its partners, thereby possibly disturbing and rewiring the interactome. Similarly, disease-associated mutations in sequence motifs of IDRs may rewire the motif-based interactomes (34).

Here, we analyzed two missense variants in biochemical and structural details because of our specific interests in the αα-hubs. Could current large-scale variant analyses provide the same mechanistic understanding? A recently developed machine learning model was used to dissect effects on protein function and abundance of all missense variants in the human proteome, including Sin3A-PAH1–A126V and Sin3A-PAH1–K155E (7, 15) (Fig. 1*C*). The study by Cagiada *et al.* (15) suggested that approximately half of all missense variants leading to loss of function and disease disrupt protein stability. The large-scale approach predicted that the two mutations leading to the PAH1 variants affect function but not stability. Large-scale studies also suggest that there is a prevalence of disease-associated mutations in buried regions and an under-representation in exposed regions, revealing a differential impact of residue location on pathogenicity (15, 35). The A126 and K155 positions are evolutionarily conserved in the PAH1 domains with strong intermolecular contacts (7), explaining the predicted functional effects (Fig. 1*C*). The machine learning approach (15), however, did not predict the increased stability of PAH1–A126V, which reveals important aspects of αα-hub function. Thus, so far, machine learning approaches can provide predictions of functionally important residues in disease-associated proteins, thereby justifying detailed experimental studies that lead to improved mechanistic understandings, as presented in this study.

## Experimental procedures

### Bioinformatics analyses

AlphaFold3 (36) was used to make models of the interactions between PAH1_119–189_ and Tet1_877–910_ or SAP25_135–160_, and PAH1_115–212_ alone. Five models were generated for each complex, and the top-ranked model for each complex was used for visualization. For the models of PAH1_115–212_ alone, all models were visualized. The models and other protein structures were visualized using the PyMOL Molecular Graphics System, version 3.0 (Schrödinger, LLC). Mutagenesis of AlphaFold3 models was made using the Mutagenesis Wizard integrated into PyMOL.

We extracted data from Cagiada *et al.* (15) using the Colab notebook (https://colab.research.google.com/github/KULL-Centre/_2024_cagiada-jonsson-func/blob/main/Download_predictions.ipynb) by entering the UniProt accession number for Sin3A (Q96ST3). From the extracted dataset, the ESM-1b and ESM-IF predictions were plotted for all mutations in the PAH1 domain using GraphPad Prism (GraphPad Software, Inc). AIUPred (37) was used with default settings for disorder predictions.

### Protein expression and purification

The DNA sequence corresponding to His-*Hs*PAH1_115–212_ was bought from Addgene in a pL01 plasmid and transformed into BL21 (DE3) cells. The cultures were grown in Luria–Bertani medium at 37 °C until an absorbance of 0.6 at 600 nm. Protein expression was induced by 0.5 mM IPTG and incubated overnight at 16 °C. Cells were harvested by centrifugation (7000*g*) for 15 min at 4 °C. The pellet was resuspended in 40 ml of wash buffer (50 mM Na_2_HPO_4_/NaH_2_PO_4_, pH 7.5, 300 mM NaCl). The cells were lysed using sonication, and the sonicate was centrifuged (17,000*g*) at 4 °C for 15 min. The soluble fraction was incubated in an immobilized metal affinity chromatography (IMAC) step with 2 to 5 ml TALON Superflow (Cytiva) resin (calibrated in wash buffer) for 1 h at 4 °C on rotation. After a wash step of 10 column volumes, the protein was eluted with 50 mM Na_2_HPO_4_/NaH_2_PO_4_, pH 7.5, 300 mM NaCl, and 150 mM imidazole. The sample was dialyzed against 2 l 20 mM Tris–HCl, pH 7.5 buffer, overnight at 4 °C. After dialysis, the sample was cleaved using tobacco etch virus protease with a molar ratio of 1:10 of enzyme to protein with 5 mM DTT and 1 mM EDTA at 4 °C overnight. After cleaving, a second dialysis step in 20 mM Tris–HCl, pH 7.5, 100 mM NaCl, followed by a second IMAC step carried out with similar conditions as the first, where the PAH1_115–212_-containing flow-through was collected. The sample was concentrated using an Amicon Ultra 3 kDa molecular weight cutoff centrifugal filter (Merck) and run on a Superdex 75 Increase 10/300 GL column (Cytiva) equilibrated with the experimental buffer of choice.

Using mutagenesis, the pL01 plasmid containing His-*Hs*PAH1_115–212_ was mutated to obtain His-*Hs*PAH1_115–212, A126V_ and His-*Hs*PAH1_115–212, K155E_ containing plasmids. The expression and purification were similar to His-*Hs*PAH1_115–212_.

pET24A plasmids containing the sequences corresponding to HsPAH1_119–189_, HsPAH1_119–189, A126V_ and HsPAH1_119–189, K155E_, bought at Twist Bioscience, were expressed in BL21 (DE3) cells. The pET24A contains a sequence for an N-terminal His-SUMO tag. The cells were grown at 37 °C until an absorbance of 0.6 to 0.8 at 600 nm in M9 minimal medium with ^15^NH_4_Cl as the sole nitrogen source. Expression was induced with 0.5 mM IPTG overnight at 16 °C before cells were harvested. Lysis and IMAC steps were performed in a similar manner to His-*Hs*PAH1_115–212_. The eluate after IMAC was dialyzed into 50 mM Tris–HCl, pH 7.5, 150 mM NaCl. The His-SUMO tag was cleaved with Ulp1 protease by adding a molar ratio of 1:100 enzyme to protein, and 1 mM DTT was added. The cleavage was done overnight at 4 °C. A second IMAC step was performed similarly to the first, leaving the protein in the flow-through, followed by freeze-drying. The freeze-dried protein was resuspended in H_2_O before running a Superdex 75 Increase 10/300 GL equilibrated with the experimental buffer of choice or into (NH_4_)HCO_3_ for a second freeze-drying step to be resuspended in experimental buffer.

The sequence corresponding to *Hs*Tet1_877–910_ was bought in the pET24A plasmid from Twist Bioscience, which also creates an N-terminal His-SUMO fusion tag. The plasmid was expressed in BL21 (DE3) cells and grown at 37 °C until an absorbance of 0.6 at 600 nm. The cells were induced, harvested, and the peptide was purified using the same procedure as for PAH1_115–212_ until the cleavage of the fusion tag. Cleavage of the His-SUMO-tag was conducted using UPL1 protease with a molar ratio of 1:150, containing 1 mM DTT, overnight at 4 °C. After the second IMAC step, the sample was lyophilized and resuspended in MilliQ H_2_O and further purified on a Superdex 75 peptide 10/300 GL column (Cytiva) equilibrated with 50 mM (NH_4_)HCO_3_. Fractions with Tet1 were lyophilized and resuspended in the experimental buffer.

The longer *Hs*Tet1 peptide, Tet1_829–930_, was also expressed in a pET24A plasmid from Twist Bioscience and purified similarly to *Hs*Tet1_877–910_.

N-terminally acetylated and C-terminally amidated SAP25_135–160_ was obtained as HPLC-purified powder from TAG Copenhagen A/S with a purity above 98%.

### Isothermal titration calorimetry

ITC experiments were performed on a MicroCal ITC_200_ calorimeter (GE Healthcare). The sample cell contained 10 to 192 μM Tet1 fragments or SAP25_135–160_, whereas 100 to 409 μM PAH1_115–212_ variants were in the syringe. A ratio between cell and syringe of 1:10 was for the experiments. Buffer conditions were 20 mM Na_2_HPO_4_/NaH_2_PO_4_, pH 7.4, 100 mM NaCl, and 1 mM Tris(2-carboxyethyl)phosphine if the peptide contained cysteines. Samples were centrifuged at 17,000*g* for 10 min at 30 °C before recording the experiments. The first injection was 0.5 μl, followed by injections of 2.0 μl with a separation time of 180 s and a total of 19 injections. All ITC experiments were obtained at 30 °C and analyzed using Origin 7 software package (MicroCal, GE Healthcare), removing the first datapoint, and fitting to a one-set of sites binding model. Three recordings were made for each interaction to calculate the mean parameters.

### CD spectropolarimetry

Far-UV CD spectra were obtained using a Jasco J-815 spectropolarimeter equipped with a Peltier thermoregulation system on samples containing 13.8 to 18 μM PAH1 variant in 20 mM Na_2_HPO_4_/NaH_2_PO_4_, pH 7.0, and 10 mM NaCl at 25 °C. Far-UV CD spectra were recorded from 260 to 190 nm with a 1 mm path length. Scanning speed was 20 nm/min, with a data pitch of 0.5 nm. Each spectrum was averaged from five scans, and the spectrum of the buffer, measured using the same settings, was subtracted. The final spectrum was smoothed using the binomial method with 10 iterations.

For thermal unfolding, the protein concentration and buffer conditions were identical to those for the far-UV spectra. The signal was followed at a fixed wavelength of 222 nm in the temperature range of 20 to 95 °C with a temperature slope of 1 °C/min. The data normalized to molar ellipticity were calculated from

(1) $$\theta_{MRW\lambda }=\frac{\frac{\text{MW}}{\left(n-1\right)}\cdot \theta_{\lambda }}{\left(10\cdot l\cdot c\right)}$$

where MW is the molecular mass of the protein in Da, n is the number of residues, $\theta_{\lambda }$ is the observed ellipticity at a given wavelength, l is the path length in cm, and *c* is the concentration in g/l. To obtain the melting temperature, *T*_m_, and the change in enthalpy upon unfolding at *T*_m,_ *ΔH*_vH_, the data were analyzed using nonlinear least square fitting:

(2) $$y\left(T\right)=\left(y_{N}+m_{1}\cdot T\right)+\left(y_{D}+m_{2}\cdot T\right)\cdot \exp\frac{\frac{{\Delta H}_{vH}\left(1-\frac{T}{T_{m}}\right)}{R\cdot T}}{1+\exp\frac{{\Delta H}_{vH}\left(1-\frac{T}{T_{m}}\right)}{R\cdot T}}$$

y(T) is the optical property at temperature, *T* (in Kelvin), $y_{N}$ and $y_{D}$ are optical properties of the native and denatured states, which are assumed to be linear (38). *m*_1_ and *m*_2_ are constants describing the temperature dependence of the signal, whereas *R* is the gas constant (J/mol·K). GraphPad Prism 10.1.1 was used during fitting.

### Nano differential scanning fluorimetry

NanoDSF was performed on a Prometheus NT.48 system (Nanotemper Technologies). Protein concentrations were determined by a Nanodrop Spectrophotometer ND-1000 using the extinction coefficient 4470 M^-1^ cm^-1^. A total of 21 to 22 samples of 40 μl in GuHCl range of 0.2 to 6.5 M were mixed from two protein stocks in a buffer containing 50 mM Na_2_HPO_4_/NaH_2_PO_4_, pH 7.0, 150 mM NaCl: one without denaturant and one with 8 M GuHCl, both with 60 μM protein. Upon excitation at 280 nm, fluorescence emission at 330 and 350 nm was measured in a temperature range of 20 to 95 °C.

The data obtained from the nanoDSF were fitted to a two-dimensional model based on a two-step denaturation using Equation 3 (24):

(3) $$y\left(T,\left[x\right]\right)=\frac{\;y_{N}\left(T,\left[x\right]\right)+y_{D}\left(T,\left[x\right]\right)\;\exp^{\frac{-\left({\Delta H}_{m}\left(1-\frac{T}{T_{m}}\right)+{\Delta C}_{p}\left(\;T-T_{m}-T\;\ln\left(\frac{T}{T_{m}}\right)\;\right)-\left[x\right]\;\left(m+m_{1}\Delta T+m_{2}{\Delta T}^{2}\right)\right)}{RT}}}{\;1+\exp^{\frac{-\left({\Delta H}_{m}\left(1-\frac{T}{T_{m}}\right)+{\Delta C}_{p}\left(\;T-T_{m}-T\;\ln\left(\frac{T}{T_{m}}\right)\;\right)-\left[x\right]\;\left(m+m_{1}\Delta T+m_{2}{\Delta T}^{2}\right)\right)}{RT}}\;}$$

Equation 3 represents the global fit that considers both thermal and chemical denaturation. ${\Delta H}_{m}$ is the change in enthalpy at *T*_m_, ${\Delta C}_{p}$ is the heat capacity change, where the system is assumed to be independent of temperature. *m*, *m*_1_, and *m*_2_ describe the *m* value at changing denaturant concentrations. *m*_1_ and *m*_2_ were fixed to zero.

$y_{N}$ and $y_{D}$ describe the pre- and post-transition baselines, respectively.

(4) $$y_{N}\left(T,\left[x\right]\right)=y_{N}\left(T_{ref}\right)+a_{0}\cdot \Delta T+c_{0}\cdot \left[x\right]$$

(5) $$y_{D}\left(T,\left[x\right]\right)={y_{D}\left(T_{ref}\right)+a}_{1}\cdot \Delta T+b_{1}\cdot {\Delta T}^{2}+c_{1}\cdot \left[x\right]$$

With $a_{0}$, $a_{1}$, $b_{1}$, $c_{0}$, and $c_{1}$ being parameters describing the temperature and denaturant dependence of the fluorescence signal, and ΔT = T - $T_{ref}$, where $T_{ref}$ is set to 298 K.

The thermodynamic values ${\Delta H}_{m}$, ${\Delta C}_{p}$, *T*_m_, *m*, *m*_1_, and *m*_2_ established from Equation 3, *m*_1_ and *m*_2_ were fixed to zero, were used in Equation 6 to estimate the Gibbs free-energy change of protein unfolding (24):

(6) $$\Delta G\left(T,\left[x\right]\right)={\Delta H}_{m}\left(1-\frac{T}{T_{m}}\right)+{\Delta C}_{p}\left(\;T-T_{m}-T\;\ln\left(\frac{T}{T_{m}}\right)\;\right)-\left[x\right]\;\left(m+m_{1}\Delta T+m_{2}{\Delta T}^{2}\right)$$

The global analysis was performed using a Python script by Hamborg *et al.*, 2020, providing the thermodynamic parameters (24, 39).

### NMR spectroscopy

Sets of ^1^H–^15^N-HSQCs were recorded on ^15^N-labeled PAH1_119–189_–wt and mutants using Bruker AVANCE 600, 750, or 800 MHz (^1^H) spectrometers, all equipped with a cryogenic probe, as described for the individual experiment types below. The temperature was set to 30 °C for all experiments, unless otherwise specified. The buffer conditions were 20 mM Na_2_HPO_4_/NaH_2_PO_4_, pH 7.4, 100 mM NaCl, 10% (v/v) D_2_O, 0.02% (w/v) NaN_3_, 0.7 mM 4,4-dimethyl-4-4-silapentane-1-sulfonic acid. In addition, 10% (v/v) of one resuspended pellet cOmplete protease inhibitor cocktail (Roche) in 1 ml 20 mM Na_2_HPO_4_/NaH_2_PO_4_, pH 7.4, 100 mM NaCl was added to the samples. Buffer conditions were used in all NMR experiments, unless other conditions are stated.

The assignment of PAH1_119–189_ was taken from the BioMagResBank (BMRB) entry 15569 (14). ^1^H–^15^N-HSQCs were recorded of ^15^N-labeled PAH1_119–189_–wt at 15, 20, 25, and 30 °C, and at pH ranging from 6.0 to 7.2. A ^1^H–^15^N-HSQC was also recorded of the protein in 20 mM Na_2_HPO_4_/NaH_2_PO_4_, pH 6.0 (conditions used in BMRB entry: 15569). The ^1^H–^15^N-HSQCs at the different conditions stated were used to transfer the assignment from the BMRB to the spectrum with the conditions used in this study.

Longitudinal (*R*_1_) and transverse (*R*_2_) relaxation experiments were recorded on the three ^15^N-labeled PAH1_119–189_ variants using a Bruker AVANCE-III HD750 MHz (^1^H) spectrometer equipped with a cryogenic probe. The pulse sequences used were hsqct1etf3gpsi3d and hsqct2etf3gpsi3d. The relaxation delays were 20, 60, 100, 200, 400, 600, 800, and 1200 ms for *T*_*1*_ and 0, 17, 34, 68, 102, 136, 170, and 237 ms for *T*_*2*_. The interscan delay was set to 2 s. All delays were recorded in triplicate and randomly. The data were processed using NMRpipe (40) and analyzed with CcpNmr (41). Peak heights at each relaxation delay were extracted and fitted to a single exponential decay function in Prism to get the relaxation rates for each residue. For the product, R_1_R_2_, error bars were propagated by the following formula

$$\frac{\sigma_{x}}{x}=\sqrt{{\left(\frac{\sigma_{a}}{a}\right)}^{2}+{\left(\frac{\sigma_{b}}{b}\right)}^{2}}$$

{^1^H}–^15^N hetNOE experiments with pulse sequence hsqcnoef3gpsi3d were recorded on the same spectrometer with a relaxation delay of 10 s. The experiments were set up in triplicates and fitted individually.

Titrations were performed for the PAH1_119–189_ variants and Tet1_877–910_. Samples of 100 μM ^15^N-PAH1_119–189_–wt, -A126, or -K155E in the absence and presence of Tet1_877–910_ were made and ^1^H–^15^N-HSQCs recoded on samples in the following ratios; 1:0, 1:0.1; 1:0.2, 1:0.3, 1:0.4, 1:0.5, 1:0.6, 1:0.7, 1:0.8, 1:0.9, 1:1, 1:2, and 1:6 for PAH1_119–189_–wt; 1:0, 1:0.1; 1:0,2, 1:0.3, 1:0.4, 1:0.5, 1:0.6, 1:0.7, 1:0.8, 1:0.9, 1:1, and 1:2 for PAH1_119–189_-A126V; and 1:0, 1:0.1; 1:0,2, 1:0.3, 1:0.4, 1:0.5, 1:0.6, 1:0.8, 1:1, 1:1.25, 1:1.5, 1:75, 1:2, and 1:4 for PAH1_119–189_-K155E.

ZZ-exchange (42) was used to identify residues in the bound state of ^15^N-PAH1_119–189_–wt in complex with Tet1_877–910_. A sample of ^15^N-PAH1_119–189_–wt (100 μM) was made with the addition of Tet1_877–910_ to a saturation level of 50% for the recordings, where both the unbound and bound states were present. Spectra with delays of 100, 150, 200, 300, and 500 ms were recorded to manually track the connection between unbound and bound peaks of the same residues.

Amide CSPs were calculated for ^15^N-PAH1_119–189_–wt in the absence and presence of Tet1_877–910_ using Equation 7 (43):

(7) ΔδNH=(ΔδH°1)2+(0.154·ΔδN°15)2

### Analytical SEC

Analytical SEC was run on a Superdex 75 Increase 10/300 GL column (Cytiva) equilibrated in 20 mM Na_2_HPO_4_/NaH_2_PO_4_, pH 7.4, 100 mM NaCl buffer. The PAH1_119–189_ variants were purified as previously described. Aliquots of 70 to 300 μM protein samples in 0.2 ml sample volumes were applied for analytical SEC on a 0.5 ml sample loop and run with a flow rate of 0.3 ml/min. A series of standard proteins was run with analytical SEC using Gel Filtration Markers Kit MWGF70 (Sigma–Aldrich). The void volume (V_0_) was determined by blue dextran run under the conditions described for the PAH1_119–189_ variants, and the proteins albumin (66 kDa) and cytochrome *C* (12.4 kDa) were mixed, and carbonic anhydrase (29 kDa) and aprotinin (6.5 kDa) were mixed and run in pairs in their recommended protein concentrations to obtain their elution volumes (*V*_e_). The known molecular weights were plotted on a semilogarithmic scale against the *V*_e_/*V*_0_ and fitted to an exponential function in Prism.

(8) $$\text{MW}=\left(2077+0.4012\right){\times e}^{\left(-2.75\times V_{e}/V_{0}\right)}-0.4012$$

The molecular weights of the PAH1 variants were calculated from this to be PAH1_119–189_-wt_MW_ = 14.2 kDa, PAH1_119–189_-A126V_MW_ = 13.9 kDa, and PAH1_119–189_-K155E_MW_ = 14.2 kDa and plotted with the standard curve.

## Data availability

All data described are contained within the article or the supporting information file. Raw data will be made available upon request to the corresponding author. The assigned backbone chemical shifts have been deposited in the BMRB under the accession codes 53414, PAH1_119–189_-wt; 53416, PAH1_119–189_-A126V; and 53415, PAH1_119–189_-K155E.

## Supporting information

This article contains supporting information (14, 36).

## Conflict of interest

The authors declare that they have no conflicts of interest with the contents of this article.

## References

[1] Adams G.E., Chandru A., Cowley S.M. (2018). Co-repressor, co-activator and general transcription factor: the many faces of the Sin3 histone deacetylase (HDAC) complex. Biochem. J..

[2] Ooi L., Wood I.C. (2007). Chromatin crosstalk in development and disease: lessons from REST. Nat. Rev. Genet..

[3] Alshehri A., Baker I.-M., English D.M., Fairall L., Collins M.O., Schwabe J.W.R. (2025). Mutations on the surface of HDAC1 reveal molecular determinants of specific complex assembly and their requirement for gene regulation. Nucleic Acids Res..

[4] Due A.D., Davey N.E., Thomasen F.E., Morffy N., Prestel A., Brakti I. (2025). Hierarchy in regulator interactions with distant transcriptional activation domains empowers rheostatic regulation. Protein Sci..

[5] Staby L., O’Shea C., Willemoës M., Theisen F., Kragelund B.B., Skriver K. (2017). Eukaryotic transcription factors: paradigms of protein intrinsic disorder. Biochem. J..

[6] Már M., Nitsenko K., Heidarsson P.O. (2023). Multifunctional intrinsically disordered regions in transcription factors. Chemistry.

[7] Bugge K., Staby L., Salladini E., Falbe-Hansen R.G., Kragelund B.B., Skriver K. (2021). αα-Hub domains and intrinsically disordered proteins: a decisive combo. J. Biol. Chem..

[8] Bugge K., Staby L., Kemplen K.R., O’Shea C., Bendsen S.K., Jensen M.K. (2018). Structure of radical-induced cell Death1 hub domain reveals a common αα-Scaffold for disorder in transcriptional networks. Structure.

[9] Friis Theisen F., Salladini E., Davidsen R., Jo Rasmussen C., Staby L., Kragelund B.B. (2022). αα-hub coregulator structure and flexibility determine transcription factor binding and selection in regulatory interactomes. J. Biol. Chem..

[10] Bernstein B.E., Tong J.K., Schreiber S.L. (2000). Genomewide studies of histone deacetylase function in yeast. Proc. Natl. Acad. Sci. U. S. A..

[11] Latypova X., Vincent M., Mollé A., Adebambo O.A., Fourgeux C., Khan T.N. (2021). Haploinsufficiency of the Sin3/HDAC corepressor complex member SIN3B causes a syndromic intellectual disability/autism spectrum disorder. Am. J. Hum. Genet..

[12] Verza F.A., Das U., Fachin A.L., Dimmock J.R., Marins M. (2020). Roles of histone deacetylases and inhibitors in anticancer therapy. Cancers (Basel).

[13] Balasubramanian M., Dingemans A.J.M., Albaba S., Richardson R., Yates T.M., Cox H. (2021). Comprehensive study of 28 individuals with SIN3A-related disorder underscoring the associated mild cognitive and distinctive facial phenotype. Eur. J. Hum. Genet..

[14] Sahu S.C., Swanson K.A., Kang R.S., Huang K., Brubaker K., Ratcliff K. (2008). Conserved themes in target recognition by the PAH1 and PAH2 domains of the Sin3 transcriptional corepressor. J. Mol. Biol..

[15] Cagiada M., Jonsson N., Lindorff-Larsen K. (2025). Decoding molecular mechanisms for loss-of-function variants in the human proteome. Elife.

[16] Williams K., Christensen J., Pedersen M.T., Johansen J.V., Cloos P.A.C., Rappsilber J. (2011). TET1 and hydroxymethylcytosine in transcription and DNA methylation fidelity. Nature.

[17] Chandru A., Bate N., Vuister G.W., Cowley S.M. (2018). Sin3A recruits Tet1 to the PAH1 domain via a highly conserved Sin3-Interaction domain. Sci. Rep..

[18] Goswami P., Banks C.A.S., Thornton J., Bengs B.D., Sardiu M.E., Florens L. (2024). Distinct regions within SAP25 recruit O-Linked glycosylation, DNA demethylation, and ubiquitin ligase and hydrolase activities to the Sin3/HDAC complex. J. Proteome Res..

[19] Dahiya N.R., Leibovitch B.A., Kadamb R., Bansal N., Waxman S. (2022). The Sin3A/MAD1 complex, through its PAH2 domain, acts as a second repressor of retinoic acid receptor beta expression in breast cancer cells. Cells.

[20] Le Guezennec X., Vermeulen M., Stunnenberg H.G. (2006). Molecular characterization of Sin3 PAH-domain interactor specificity and identification of PAH partners. Nucleic Acids Res..

[21] Kumar G.S., Xie T., Zhang Y., Radhakrishnan I. (2011). Solution structure of the mSin3A PAH2-Pf1 SID1 complex: a Mad1/Mxd1-like interaction disrupted by MRG15 in the Rpd3S/Sin3S complex. J. Mol. Biol..

[22] Brubaker K., Cowley S.M., Huang K., Loo L., Yochum G.S., Ayer D.E. (2000). Solution structure of the interacting domains of the Mad-Sin3 complex: implications for recruitment of a chromatin-modifying complex. Cell.

[23] Nomura M., Uda-Tochio H., Murai K., Mori N., Nishimura Y. (2005). The neural repressor NRSF/REST binds the PAH1 domain of the Sin3 corepressor by using its distinct short hydrophobic helix. J. Mol. Biol..

[24] Hamborg L., Horsted E.W., Johansson K.E., Willemoës M., Lindorff-Larsen K., Teilum K. (2020). Global analysis of protein stability by temperature and chemical denaturation. Anal. Biochem..

[25] Myers J.K., Nick Pace C., Martin Scholtz J. (1995). Denaturant *m* values and heat capacity changes: relation to changes in accessible surface areas of protein unfolding. Protein Sci..

[26] Kneller J.M., Lu M., Bracken C. (2002). An effective method for the discrimination of motional anisotropy and chemical exchange. J. Am. Chem. Soc..

[27] Skriver K., Theisen F.F., Kragelund B.B. (2023). Conformational entropy in molecular recognition of intrinsically disordered proteins. Curr. Opin. Struct. Biol..

[28] Bugge K., Brakti I., Fernandes C.B., Dreier J.E., Lundsgaard J.E., Olsen J.G. (2020). Interactions by disorder - a matter of context. Front. Mol. Biosci..

[29] Theisen F.F., Staby L., Tidemand F.G., O’Shea C., Prestel A., Willemoës M. (2021). Quantification of Conformational Entropy Unravels effect of disordered flanking Region in coupled folding and binding. J. Am. Chem. Soc..

[30] Hasan T., Ali M., Saluja D., Singh L.R. (2015). pH might play a role in regulating the function of paired amphipathic helices domains of human Sin3B by altering structure and thermodynamic stability. Biochemistry (Moscow).

[31] Kjaergaard M., Teilum K., Poulsen F.M. (2010). Conformational selection in the molten globule state of the nuclear coactivator binding domain of CBP. Proc. Natl. Acad. Sci. U. S. A..

[32] Berlow R.B., Martinez-Yamout M.A., Dyson H.J., Wright P.E. (2019). Role of backbone dynamics in modulating the interactions of disordered ligands with the TAZ1 domain of the CREB-Binding protein. Biochemistry.

[33] Alhindi T., Zhang Z., Ruelens P., Coenen H., Degroote H., Iraci N. (2017). Protein interaction evolution from promiscuity to specificity with reduced flexibility in an increasingly complex network. Sci. Rep..

[34] Kliche J., Simonetti L., Krystkowiak I., Kuss H., Diallo M., Rask E. (2024). Proteome-scale characterisation of motif-based interactome rewiring by disease mutations. Mol. Syst. Biol..

[35] Savojardo C., Manfredi M., Martelli P.L., Casadio R. (2021). Solvent accessibility of residues undergoing pathogenic variations in humans: from protein structures to protein sequences. Front. Mol. Biosci..

[36] Abramson J., Adler J., Dunger J., Evans R., Green T., Pritzel A. (2024). Accurate structure prediction of biomolecular interactions with AlphaFold 3. Nature.

[37] Erdős G., Dosztányi Z. (2024). AIUPred: combining energy estimation with deep learning for the enhanced prediction of protein disorder. Nucleic Acids Res..

[38] Haque M.A., Kaur P., Islam A., Hassan M.I. (2022). Application of circular dichroism spectroscopy in studying protein folding, stability, and interaction. Adv. Protein Mol. Struct. Biol. Methods.

[39] Magnusson A.O., Szekrenyi A., Joosten H.J., Finnigan J., Charnock S., Fessner W.D. (2019). nanoDSF as screening tool for enzyme libraries and biotechnology development. FEBS J..

[40] Delaglio F., Grzesiek S., Vuister G.W., Zhu G., Pfeifer J., Bax A. (1995). NMRPipe: a multidimensional spectral processing system based on UNIX pipes. J. Biomol. NMR..

[41] Vranken W.F., Boucher W., Stevens T.J., Fogh R.H., Pajon A., Llinas M. (2005). The CCPN data model for NMR spectroscopy: development of a software pipeline. Proteins.

[42] Vallurupalli P., Kay L.E. (2013). Probing slow chemical exchange at carbonyl sites in proteins by chemical exchange saturation transfer NMR spectroscopy. Angew. Chem. Int. Ed..

[43] Williamson M.P. (2013). Using chemical shift perturbation to characterise ligand binding. Prog. Nucl. Magn. Reson Spectrosc..

