# Supplementary material for: Stability and interaction defects in the Sin3A-PAH1 αα-hub domain associated with loss-of-function variants
Source: J Biol Chem. 2026 Feb 26;302(4):111312. doi: 10.1016/j.jbc.2026.111312 (PMC13068551; doi:10.1016/j.jbc.2026.111312)
Supplement: Supporting information [file mmc1.pdf]

## Supporting Information

# Stability and interaction defects in the Sin3A PAH1 αα-hub domain associated with loss of function variants

Amanda D. Due[1,2,3], Sigrid Jørsboe[2], Louise T. Jensen[2], Charlotte O'Shea[2], Majken Staulund[2], Martin Willemoës[2], Birthe B. Kragelund[1,2,3], Ida M. Z. Sjøgaard[1,2*], and Karen Skriver[1,2,*]

[1] REPIN, University of Copenhagen, Copenhagen, Denmark.

[2] Linderstrøm-Lang Centre for Protein Science, University of Copenhagen, Copenhagen, Denmark.

[3] Structural Biology and NMR Laboratory, Department of Biology, University of Copenhagen, Copenhagen, Denmark.

**Running title:** *Sin3a αα-hub domain: stability and dysfunction*

**List of contents:**

**Fig. S1** AlphaFold3 models of PAH1$_{115\text{-}212}$-wt fragment

**Fig. S2** Unfolding experiments of Sin3A-PAH1 variants by denaturant at increasing temperatures

**Fig. S3** Unfolding experiments of Sin3A-PAH1 variants by temperature at increasing GuHCl concentrations

**Fig. S4** Backbone N, H$^N$-assignment of PAH1$_{119\text{-}189}$-wt

**Fig. S5** pH titration of $^{15}$N-PAH1$_{119\text{-}189}$-wt to assign the peaks under different conditions

**Fig. S6** Temperature titration of $^{15}$N-PAH1$_{119\text{-}189}$-wt to assign the peaks in different conditions

**Fig. S7** Product of $R_1$ and $R_2$ for PAH1-variants

**Fig. S8** Analytical SEC of PAH1-variants

**Fig. S9** Second and third replicate of ITC experiments of the interactions between PAH1$_{115\text{-}212}$ variants and Tet1 fragments

**Fig. S10** Second and third replicate of the ITC experiments of the interactions between PAH1$_{115\text{-}212}$ variants and SAP25$_{135\text{-}160}$

**Fig. S11** NMR titrations with PAH1$_{119\text{-}189}$ variants and Tet1$_{877\text{-}910}$

**Fig. S12** ZZ-exchange to identify the bound state of PAH1$_{119\text{-}189}$-wt in interaction with Tet1$_{877\text{-}910}$

**Fig. S13** Chemical shift perturbations of PAH1$_{119\text{-}189}$-wt upon binding to Tet1$_{870\text{-}910}$

**Fig. S14** AlphaFold3 models of PAH1$_{115\text{-}212}$-wt in complex Tet1$_{877\text{-}910}$ and SAP25$_{135\text{-}160}$

**References**

1. Abramson, J., Adler, J., Dunger, J., Evans, R., Green, T., Pritzel, A., Ronneberger, O., Willmore, L., Ballard, A. J., Bambrick, J., Bodenstein, S. W., Evans, D. A., Hung, C.-C., O'Neill, M., Reiman, D., Tunyasuvunakool, K., Wu, Z., Žemgulytė, A., Arvaniti, E., Beattie, C., Bertolli, O., Bridgland, A., Cherepanov, A., Congreve, M., Cowen-Rivers, A. I., Cowie, A., Figurnov, M., Fuchs, F. B., Gladman, H., Jain, R., Khan, Y. A., Low, C. M. R., Perlin, K., Potapenko, A., Savy, P., Singh, S., Stecula, A., Thillaisundaram, A., Tong, C., Yakneen, S., Zhong, E. D., Zielinski, M., Žídek, A., Bapst, V., Kohli, P., Jaderberg, M., Hassabis, D., and Jumper, J. M. (2024) Accurate structure prediction of biomolecular interactions with AlphaFold 3. *Nature*. 630, 493–500

2. Sahu, S. C., Swanson, K. A., Kang, R. S., Huang, K., Brubaker, K., Ratcliff, K., and Radhakrishnan, I. (2008) Conserved Themes in Target Recognition by the PAH1 and PAH2 Domains of the Sin3 Transcriptional Corepressor. *J Mol Biol*. 375, 1444–1456

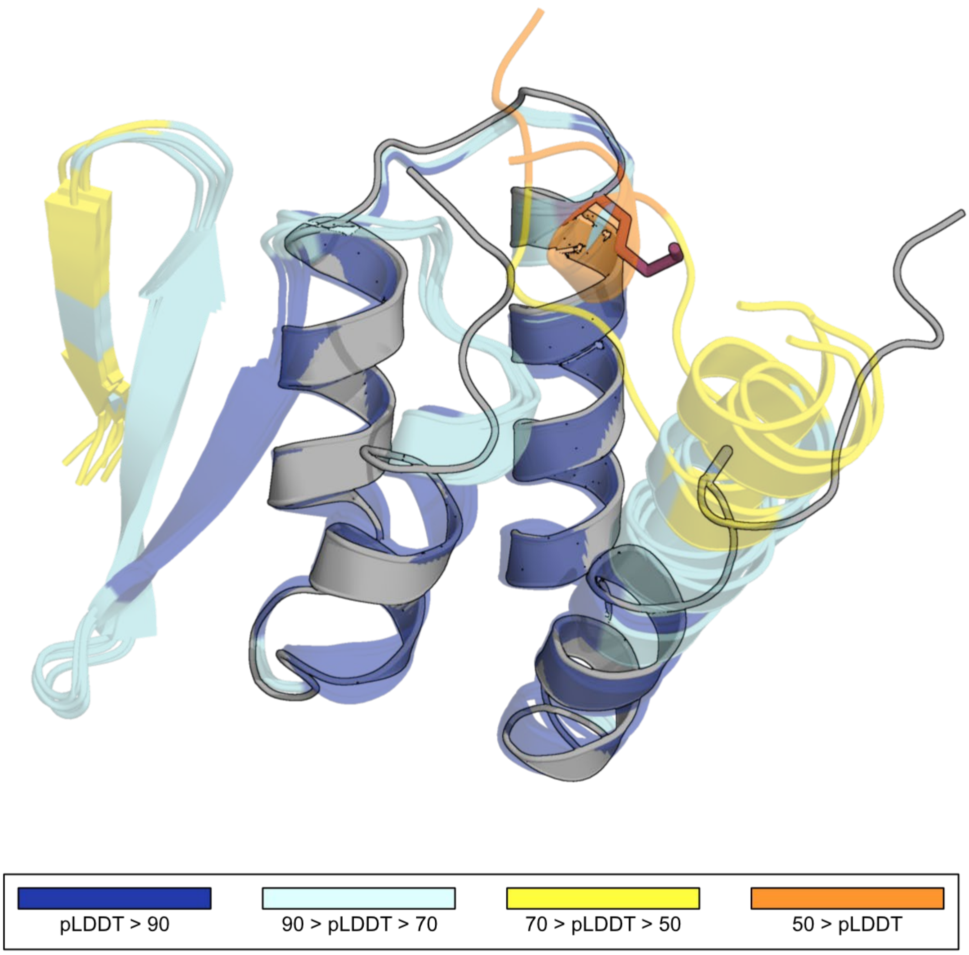


**Figure S1: AlphaFold3 models of PAH1$_{115\text{-}212}$-wt fragment.** Superimposed AlphaFold3 (1) models of PAH1$_{115\text{-}212}$ and PDB structure of PAH1$_{119\text{-}189}$ (PDB: 2rmr) (2). The PDB structure is shown in grey and AlphaFold3 models are transparent and colored based on their pLDDT score (color codes shown below). A126 (purple) and K155 (red) are shown as sticks in the PDB structure.

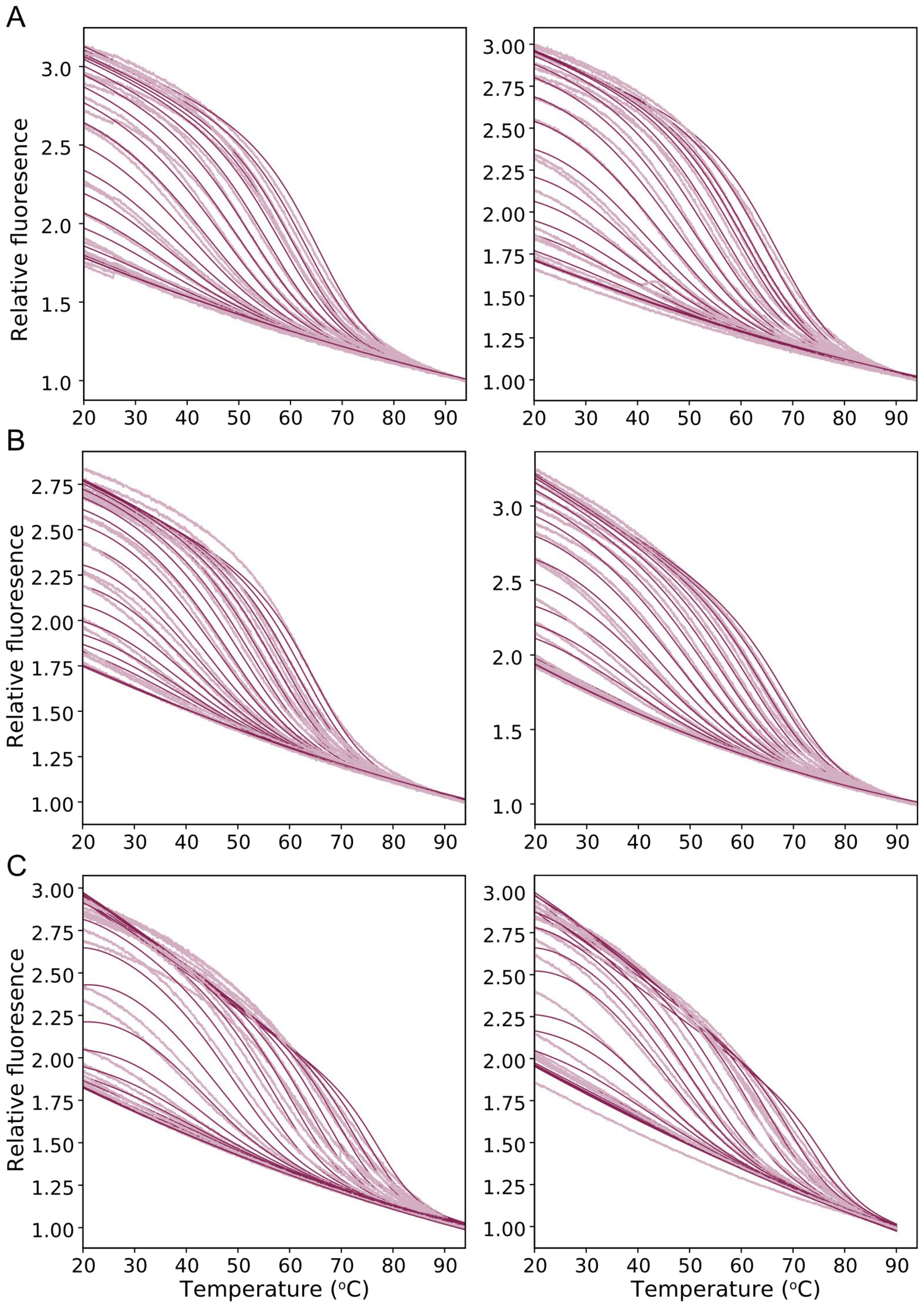


**Figure S2: Unfolding experiments of Sin3A-PAH1 variants by denaturant at increasing temperatures.** The second and third replicate of NanoDSF experiments with two-dimensional unfolding analysis of denaturant denaturation ([GuHCl] of 0.2 – 6.5 M) data by measuring internal fluorescence at 330 nm and fitting to Equation 3. (A) PAH1$_{115-212}$-wt, (B) PAH1$_{115-212}$-A126V, and (C) PAH1$_{115-212}$-K155E. The experimental data are shown in light purple, while the fits are shown as dark purple lines.

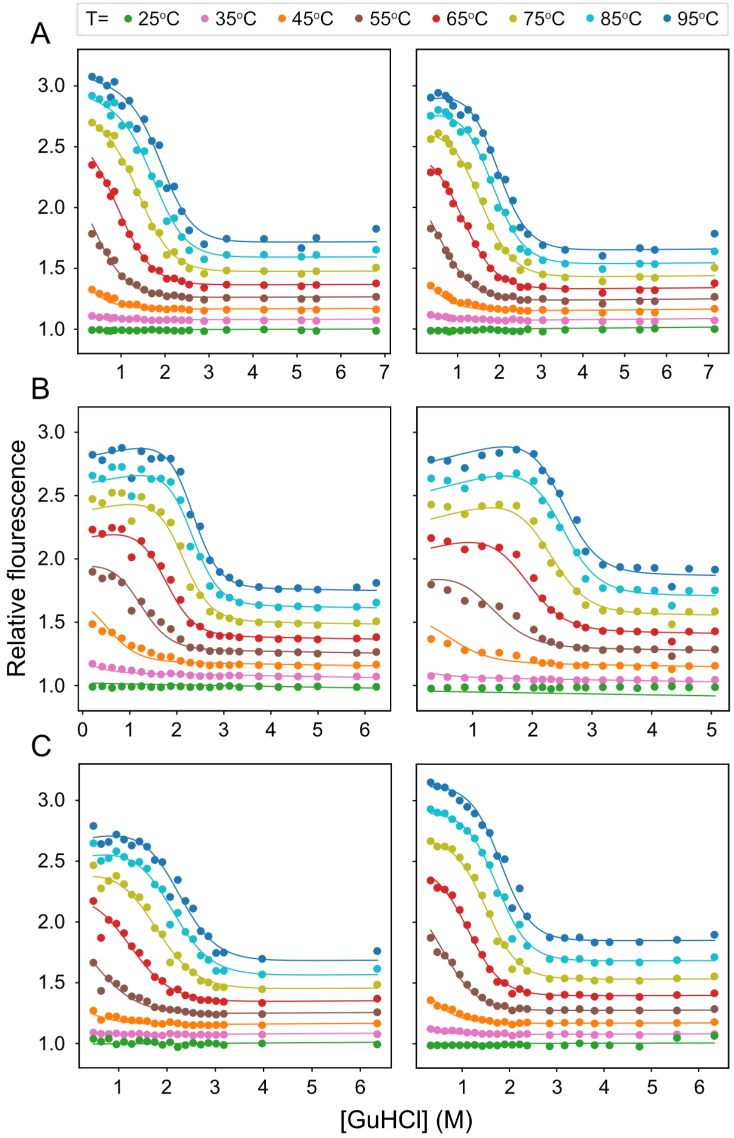


**Figure S3: Unfolding experiments of Sin3A-PAH1 variants by temperature at increasing GuHCl concentrations.** The second and third replicate of NanoDSF experiments with two-dimensional unfolding analysis of temperature denaturation data by measuring internal fluorescence at 330 nm and fitting to Equation 3. (A) PAH1$_{115\text{-}212}$-wt, (B) PAH1$_{115\text{-}212}$-A126V, and (C) PAH1$_{115\text{-}212}$-K155E. Temperatures are colored as shown above the plots. The data are shown as dots, while the fits are shown as lines.

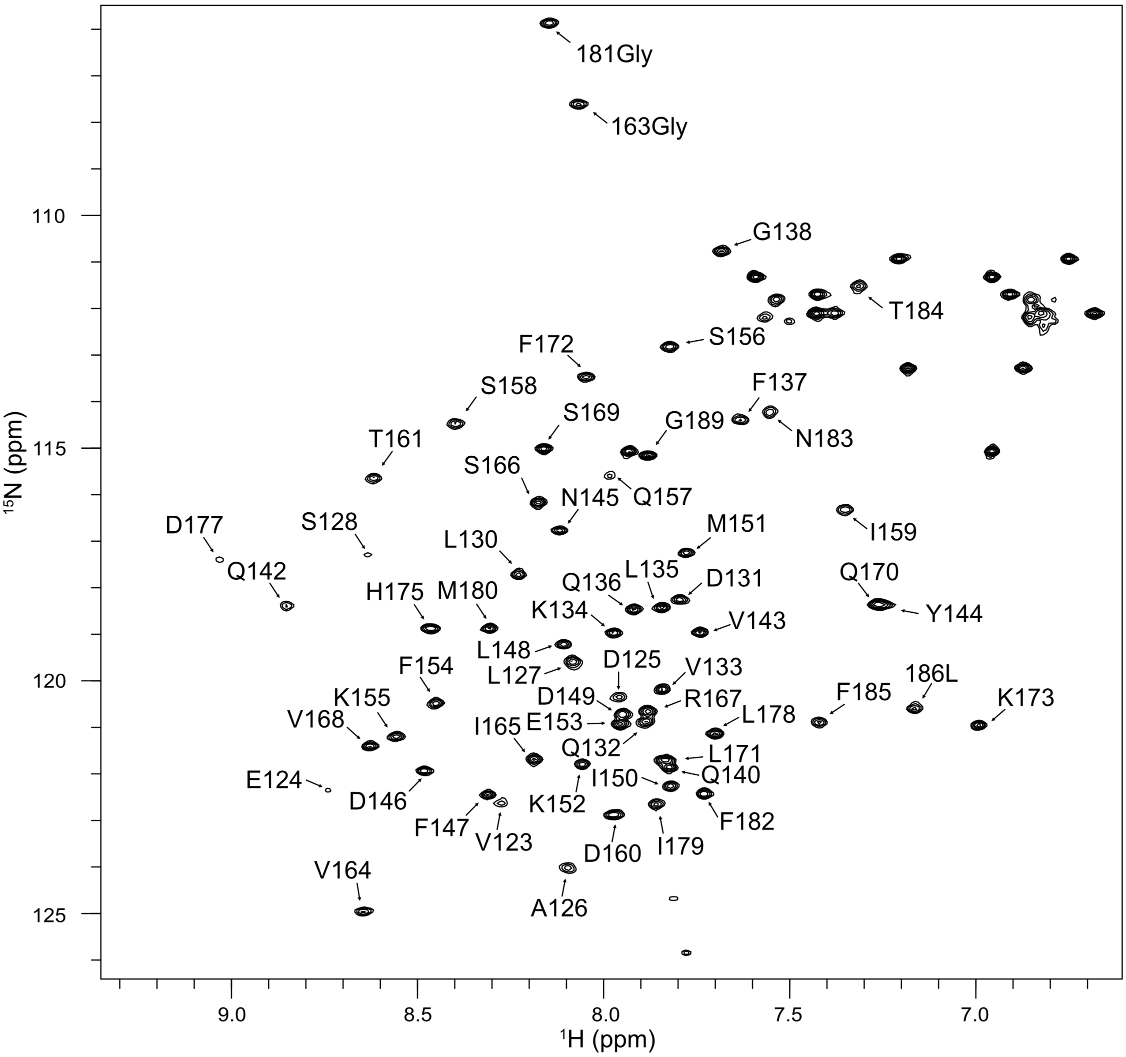


**Figure S4: Backbone N, $H^N$-assignment of $PAH1_{119\text{-}189}$-wt**. Conditions: 20 mM $Na_2HPO_4$/ $NaH_2PO_4$, pH 7.4, 100 mM NaCl at 30 °C.

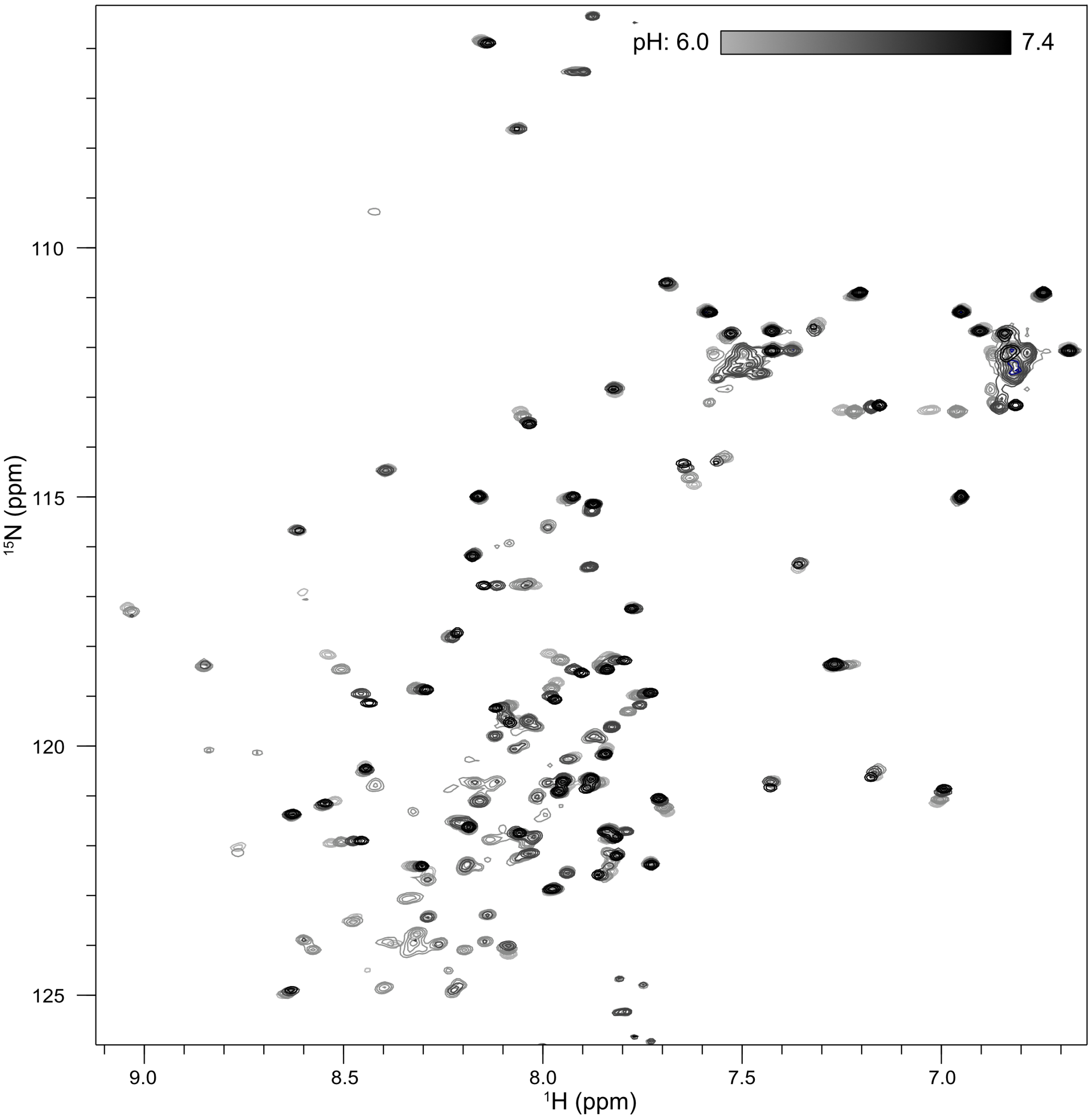


**Figure S5: pH titration of $^{15}$N-PAH1$_{119\text{-}189}$-wt to assign the peaks under different conditions.** $^{1}$H- $^{15}$N-HSQCs of $^{15}$N-PAH1$_{119\text{-}189}$ superimposed at different pH as indicated in the upper right corner of the plot.

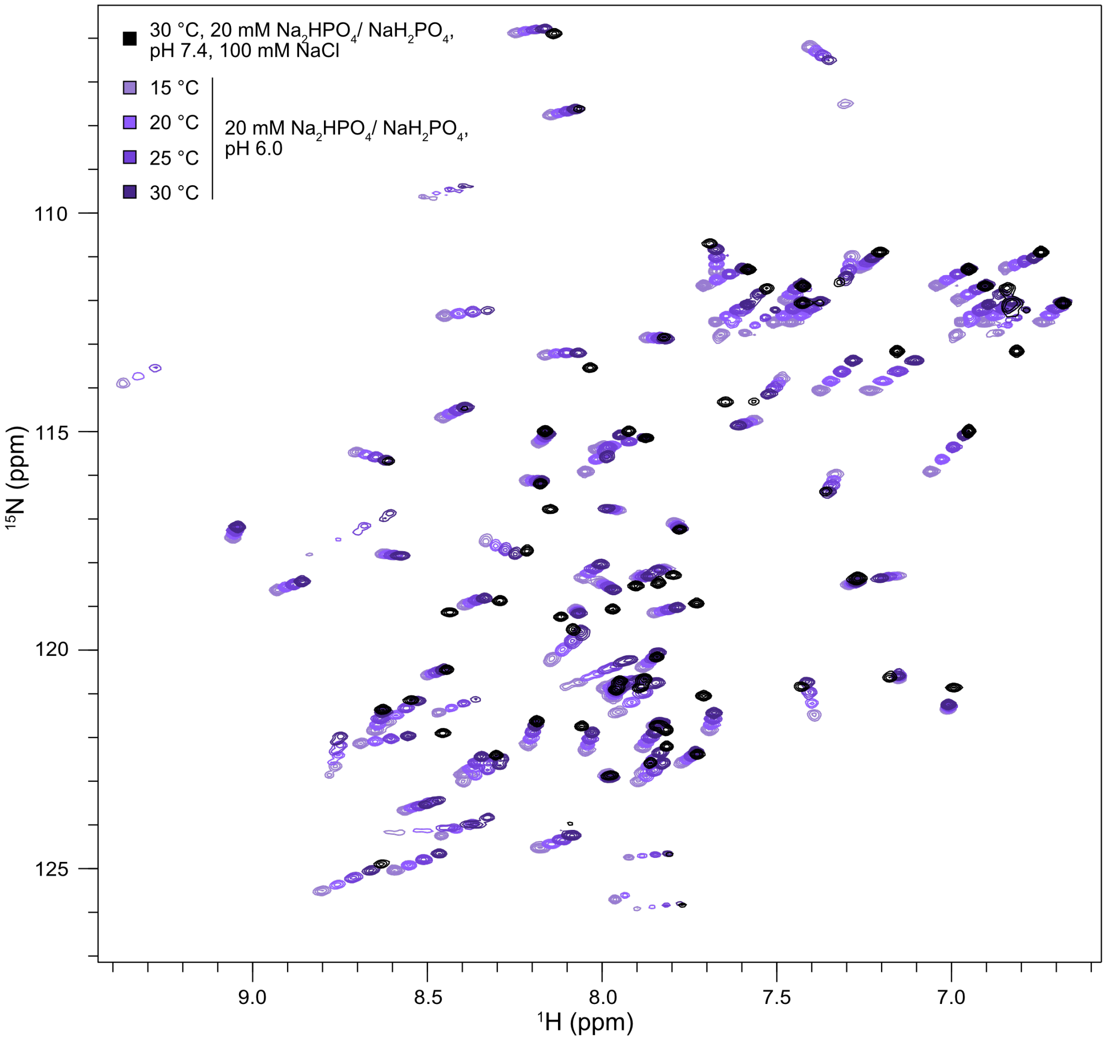


**Figure S6: Temperature titration of $^{15}$N-PAH1$_{119\text{-}189}$-wt to assign the peaks in different conditions.** $^{1}$H- $^{15}$N-HSQCs of $^{15}$N-PAH1$_{119\text{-}189}$ superimposed at different temperatures and buffer conditions as indicated in the upper left corner of the plot.

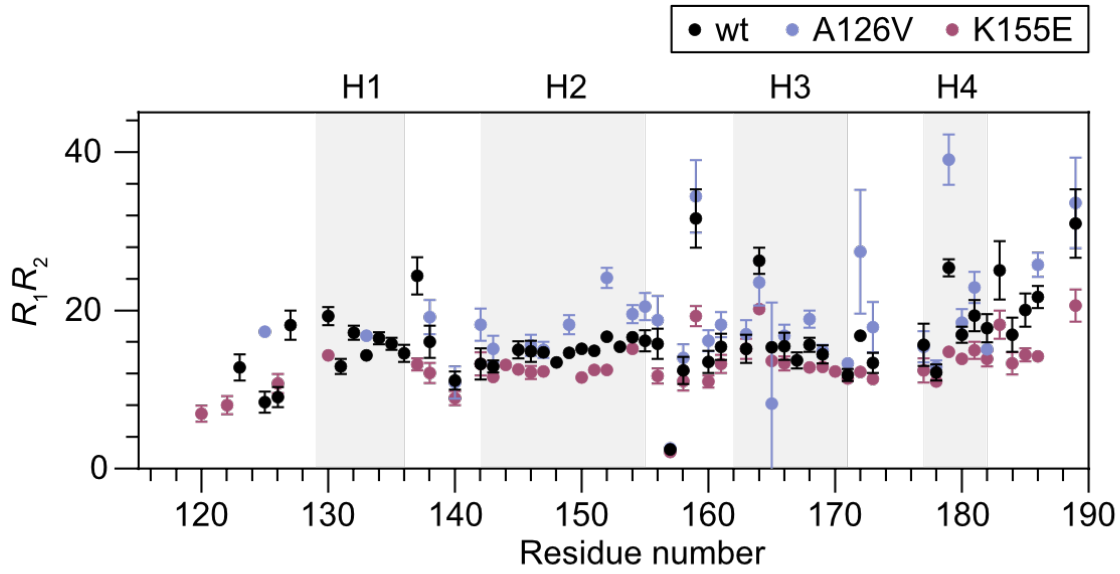


**Figure S7: Product of $R_1$ and $R_2$ for PAH1-variants**. Coloring of the dataset is shown above the plot. Marked areas in light grey represent H1-H4. Standard error of the fit is included for each residue and propagated from the SE of the individual $R_1$ and $R_2$ as described in Experimental procedures.

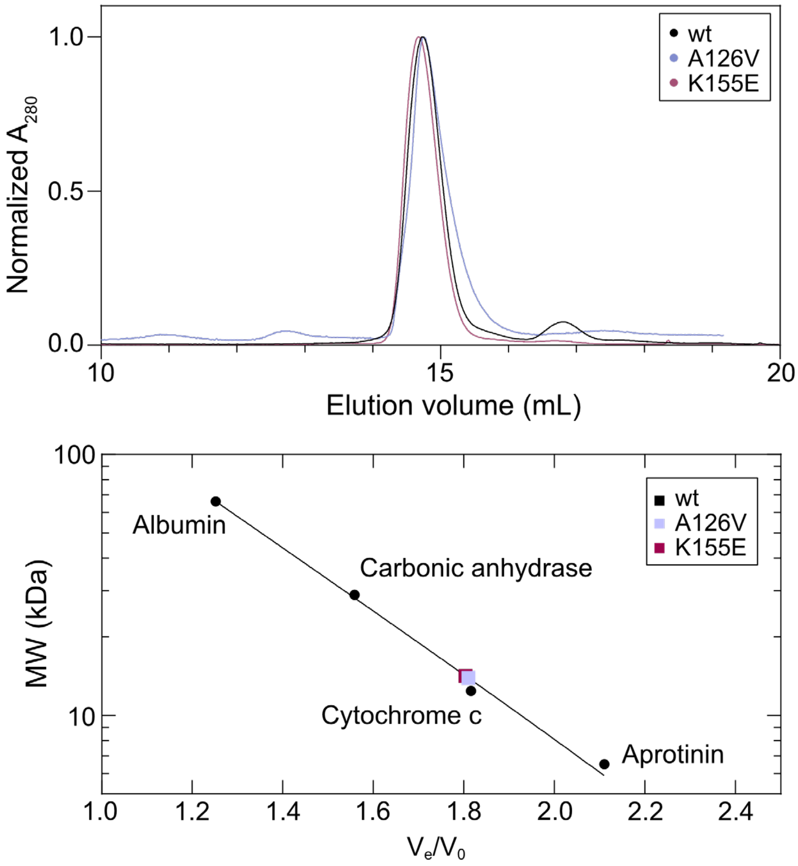


**Figure S8: Analytical SEC of PAH1-variants.** (A) Elution profile for $PAH1_{119\text{-}189}$-wt, -A126V, and -K155E on a Superdex™ 75 Increase 10/300 GL (Cytiva). The elution profiles of the proteins were normalized to their individual highest $A_{280}$. (B) Standard curve obtained with proteins from an MWGF70 Kit run on a Superdex™ 75 Increase 10/300 GL (Cytiva). PAH1 domain variants are plotted according to their $V_e/V_0$ ratio and determined MW. Coloring of the profiles as indicated in the upper right corner of the plots.

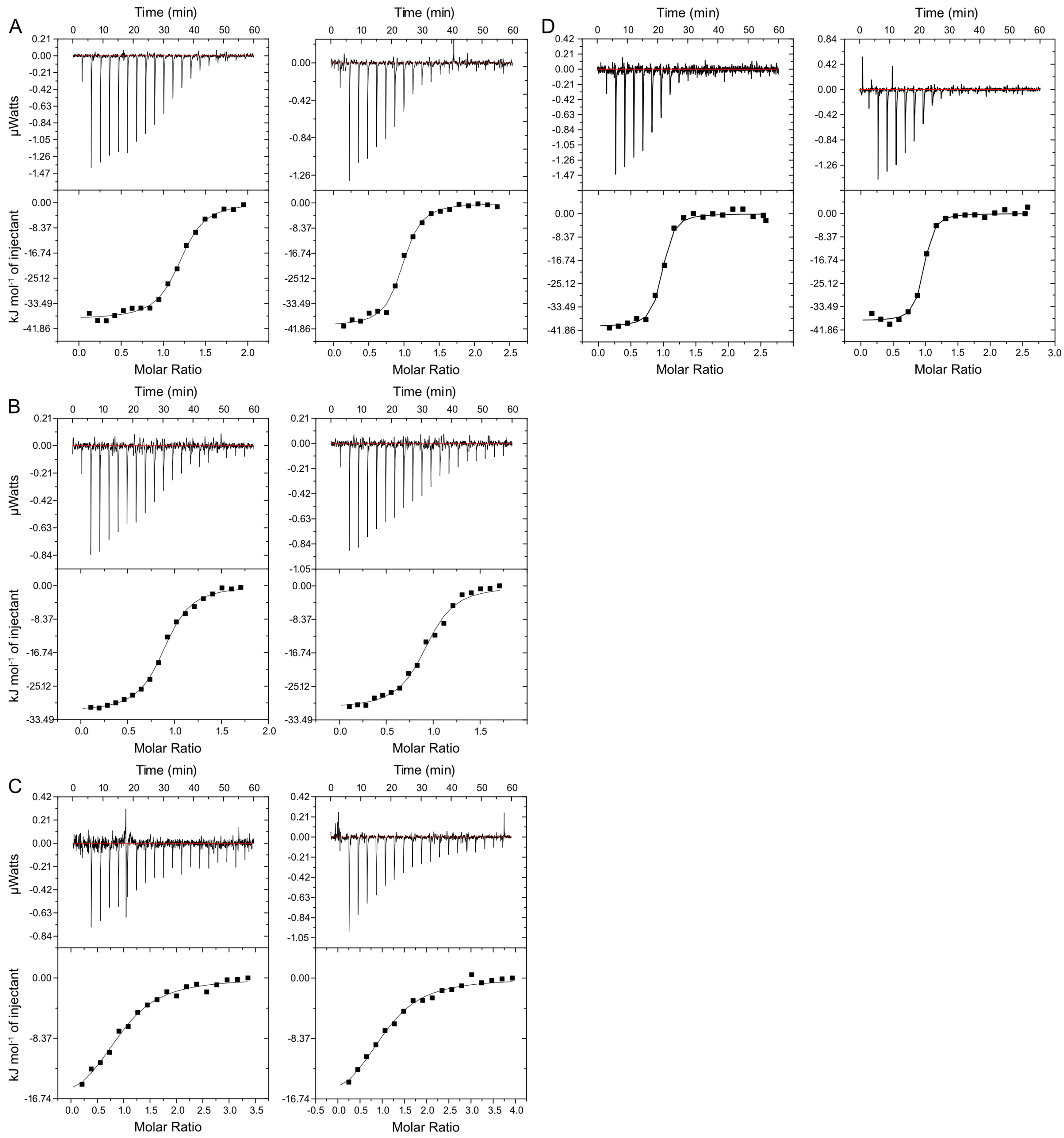


**Figure S9: Second and third replicate of ITC experiments of the interactions between PAH1$_{115\text{-}212}$ variants and Tet1 fragments.** The second and third ITC experiments were used for the calculation of mean thermodynamic parameters in Table 2 (main text) for PAH1$_{115\text{-}212}$-wt (A), PAH1$_{115\text{-}212}$-A126V (B), and PAH1$_{115\text{-}212}$-K155E (C) in interaction with Tet1$_{877\text{-}910}$, and between PAH1$_{115\text{-}212}$-wt and Tet1$_{829\text{-}930.}$ (D) .

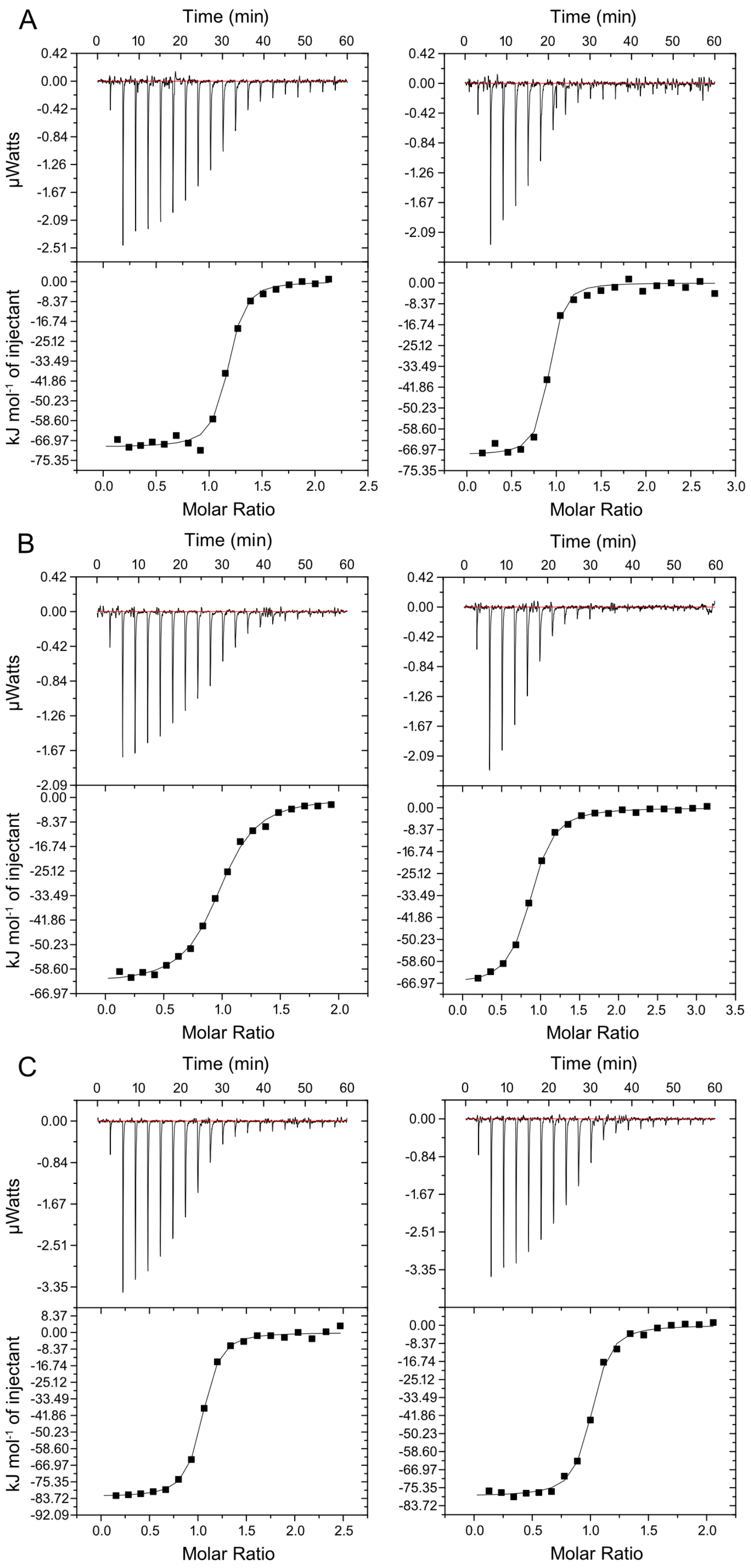


**Figure S10: Second and third replicate of the ITC experiments of the interactions between PAH1$_{115\text{-}212}$ variants and SAP25$_{135\text{-}160}$.** The second and third ITC experiments were used for the calculation of mean thermodynamic parameters in Table 2 (main text) for PAH1$_{115\text{-}212}$-wt (A), PAH1$_{115\text{-}212}$-A126V (B), and PAH1$_{115\text{-}212}$-K155E (C).

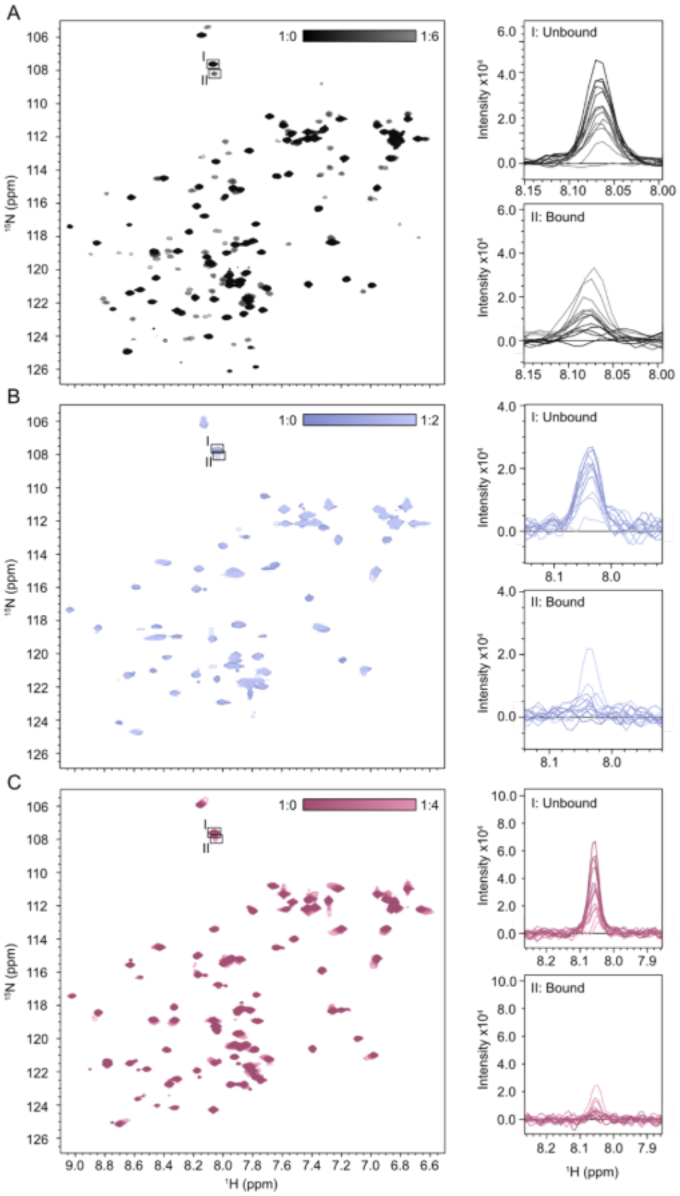


**Fig. S11: NMR titrations with PAH1$_{119\text{-}189}$ variants and Tet1$_{877\text{-}910}$.** Titrations of 100 µM $^{15}$N PAH1$_{119\text{-}189}$-wt (A), $^{15}$N PAH1$_{119\text{-}189}$-A126V (B), and $^{15}$N PAH1$_{119\text{-}189}$-K155E (C) with Tet1$_{877\text{-}910}$ in the protein ratios indicated by the gradient bar in each plot with superimposed HSQC spectra. To the right, the intensities for the unbound (top, I) and bound (bottom, II) peaks of glycine 163 are shown in the proton dimension for the titration steps for all PAH1$_{119\text{-}189}$ variants. The peak positions are indicated in the HSQCs and marked by I (unbound) and II (bound).

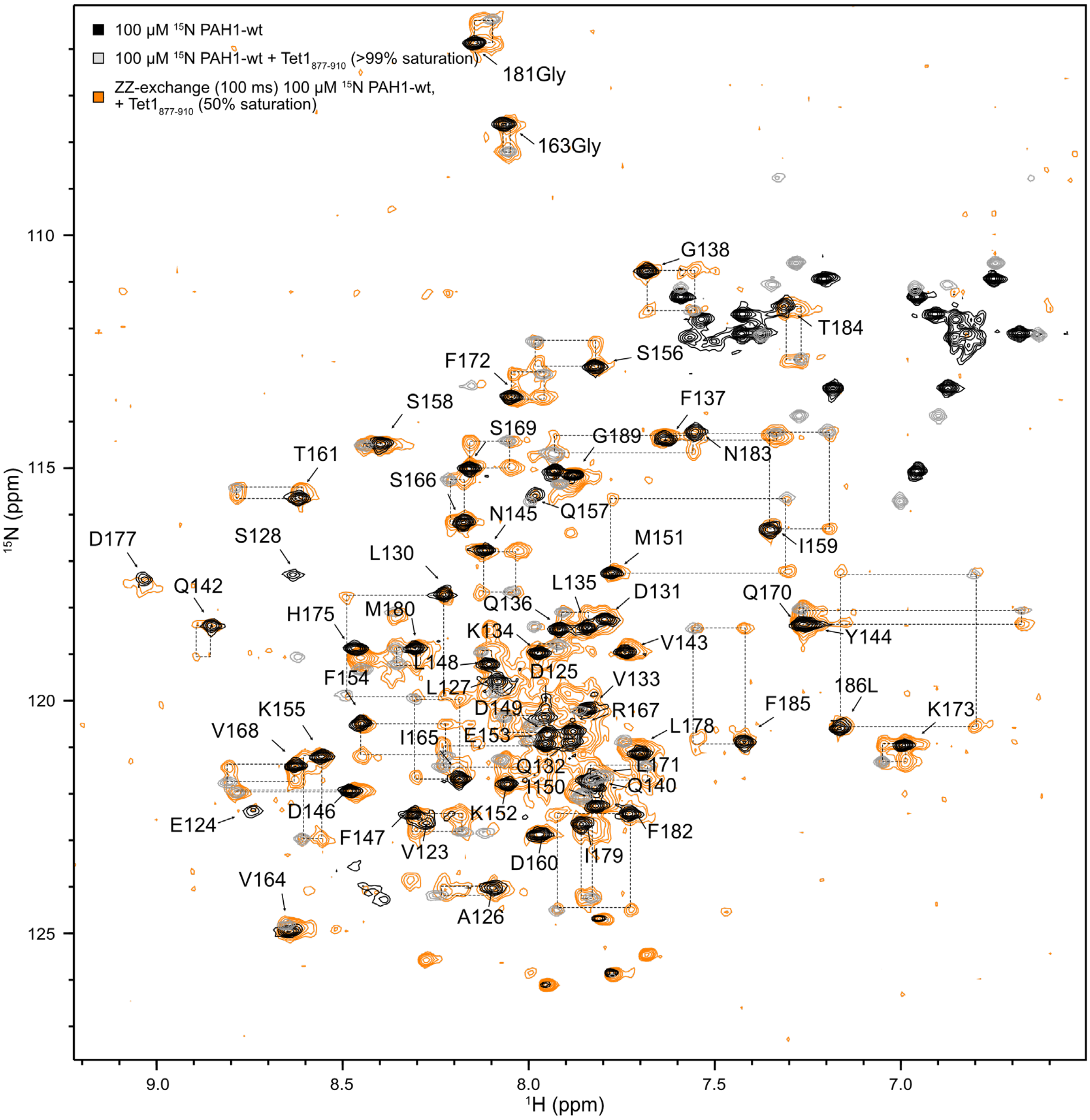


**Figure S12: ZZ-exchange to identify the bound state of PAH1$_{119-189}$-wt in interaction with Tet1$_{877-910}$.** Superimposed spectra of unbound 100 µM $^{15}$N PAH1$_{119-189}$-wt (black) with Tet1$_{877-910}$ to a saturation level of 50% (orange) and >99% (grey). ZZ-exchange with a delay of 100ms was used during the recording of the orange spectrum. The cross peaks connecting unbound and bound residues of PAH1$_{119-189}$-wt are indicated by dashed lines. Additional delays were used but are not shown in the superimposition.

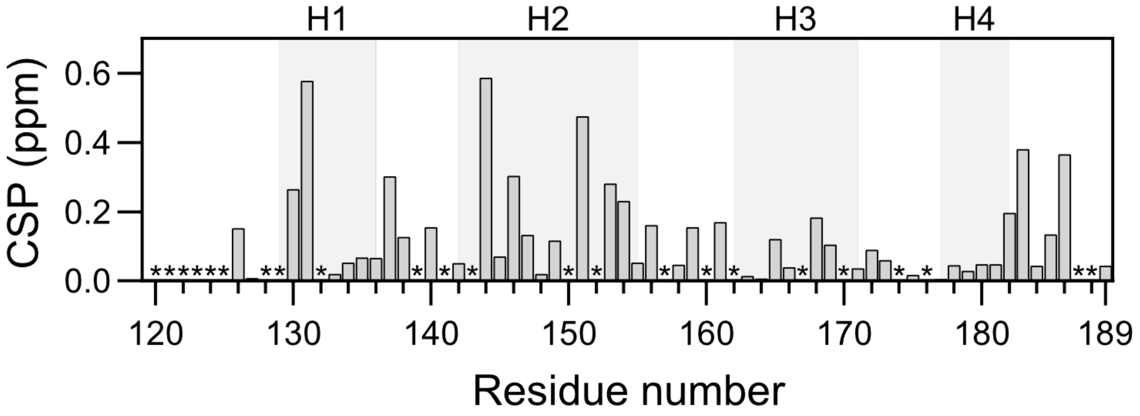


**Figure S13: Chemical shift perturbations of PAH1$_{119\text{-}189}$-wt upon binding to Tet1$_{870\text{-}910}$.** A 100µM PAH1$_{119\text{-}189}$-wt in >99% saturation of Tet1$_{870\text{-}910}$ was used to calculate the chemical shift perturbations for residues identified in the bound state using ZZ-exchange. * marks unassigned bound state residues. Grey areas represent H1-H4 in the PAH1 domain.

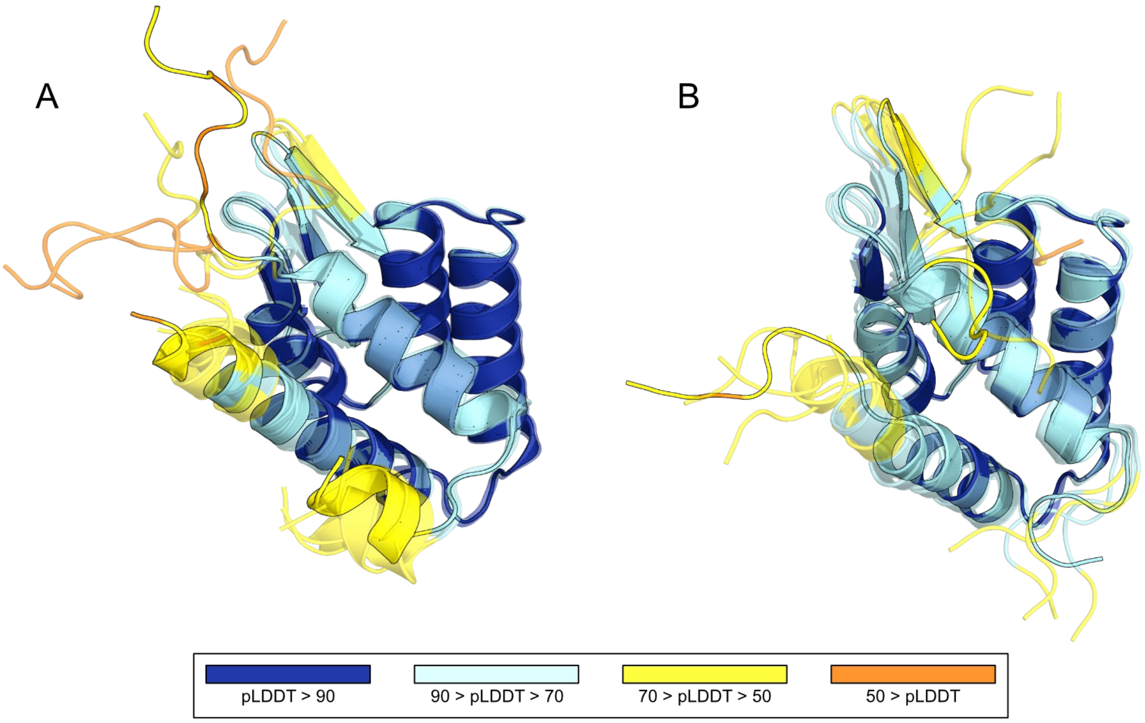


**Figure S14: AlphaFold3 models of PAH1$_{115\text{-}212}$-wt in complex with Tet1$_{877\text{-}910}$ and SAP25$_{135\text{-}160}$.** The five generated Alphafold3 models for the complexes between PAH1$_{115\text{-}212}$-wt and Tet1$_{877\text{-}910}$ (A) and SAP25$_{135\text{-}160}$ (B) (1). All models are colored based on the pLDDT score as indicated below the models. The highest-ranking model, also depicted in Fig. 5, is shown in saturated color with the four additional models being transparent.
